# Supplementary material for: Structural details of helix-mediated multimerization of the conserved region of TDP-43 C-terminal domain
Source: Nat Commun. 2025 Nov 26;16:10528. doi: 10.1038/s41467-025-65546-w (PMC12658102; doi:10.1038/s41467-025-65546-w)

Supplementary Information appendix

Structural details of helix-mediated multimerization of the conserved region of TDP-43 C-terminal domain

Azamat Rizuan[1#], Jayakrishna Shenoy[2#], Priyesh Mohanty[1], Patricia M. S. dos Passos[3], José F. Mercado Ortiz[2], Leanna Bai[2], Renjith Viswanathan[2], Julia Zaborowksy[2], Szu-Huan Wang[2], Victoria Johnson[2], Lohany D. Mamede[3], Amanda R. Titus[3], Yuna M. Ayala[3], Rodolfo Ghirlando[4], Jeetain Mittal[1,5,6*], Nicolas L. Fawzi[2*]

1. Artie McFerrin Department of Chemical Engineering, Texas A&M University, College Station, TX 77843
2. Department of Molecular Biology, Cell Biology & Biochemistry, Brown University, Providence, RI 02912
3. Edward Doisy Department of Biochemistry and Molecular Biology, Saint Louis University School of Medicine, St. Louis, Missouri
4. Laboratory of Molecular Biology, National Institute of Diabetes, Digestive and Kidney Diseases, National Institutes of Health, Bethesda, MD 20892
5. Department of Chemistry, Texas A&M University, College Station, TX 77843
6. Interdisciplinary Graduate Program in Genetics and Genomics, Texas A&M University, College Station, TX 77843

[#]equal contribution
*Correspondence:
jeetain@tamu.edu
nicolas_fawzi@brown.edu

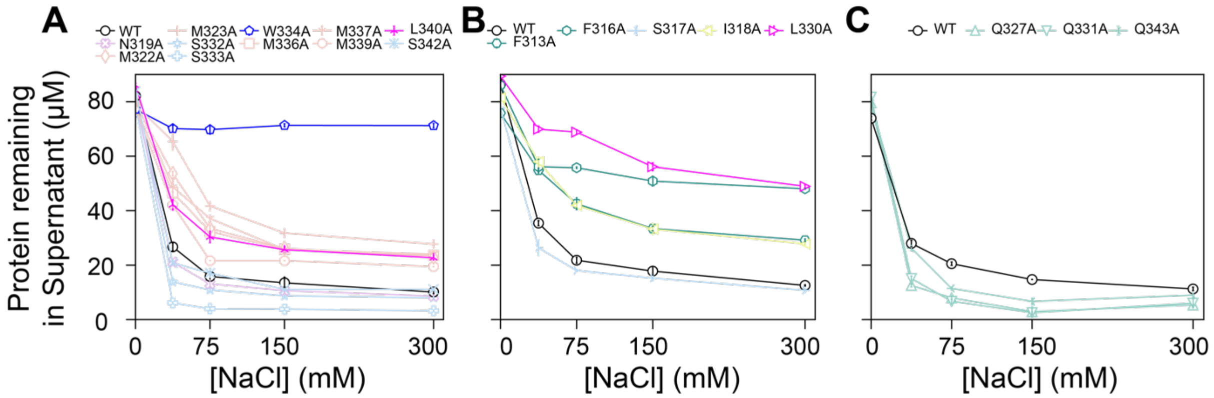

Supplementary Figure 1. Phase separation saturation concentrations of TDP-43 CTD and designed variants from alanine-scanning mutagenesis. A-C. Quantification of phase separation assay showing the protein remaining in the supernatant after phase separation for WT CTD and its single alanine substitution variants at non-alanine positions within CR and its adjacent residues measured from 0 to 300 mM NaCl from the individual experiments that were performed together. Data obtained from n = 3 technical replicates, mean ± SD. Source data are provided as a Source Data file.

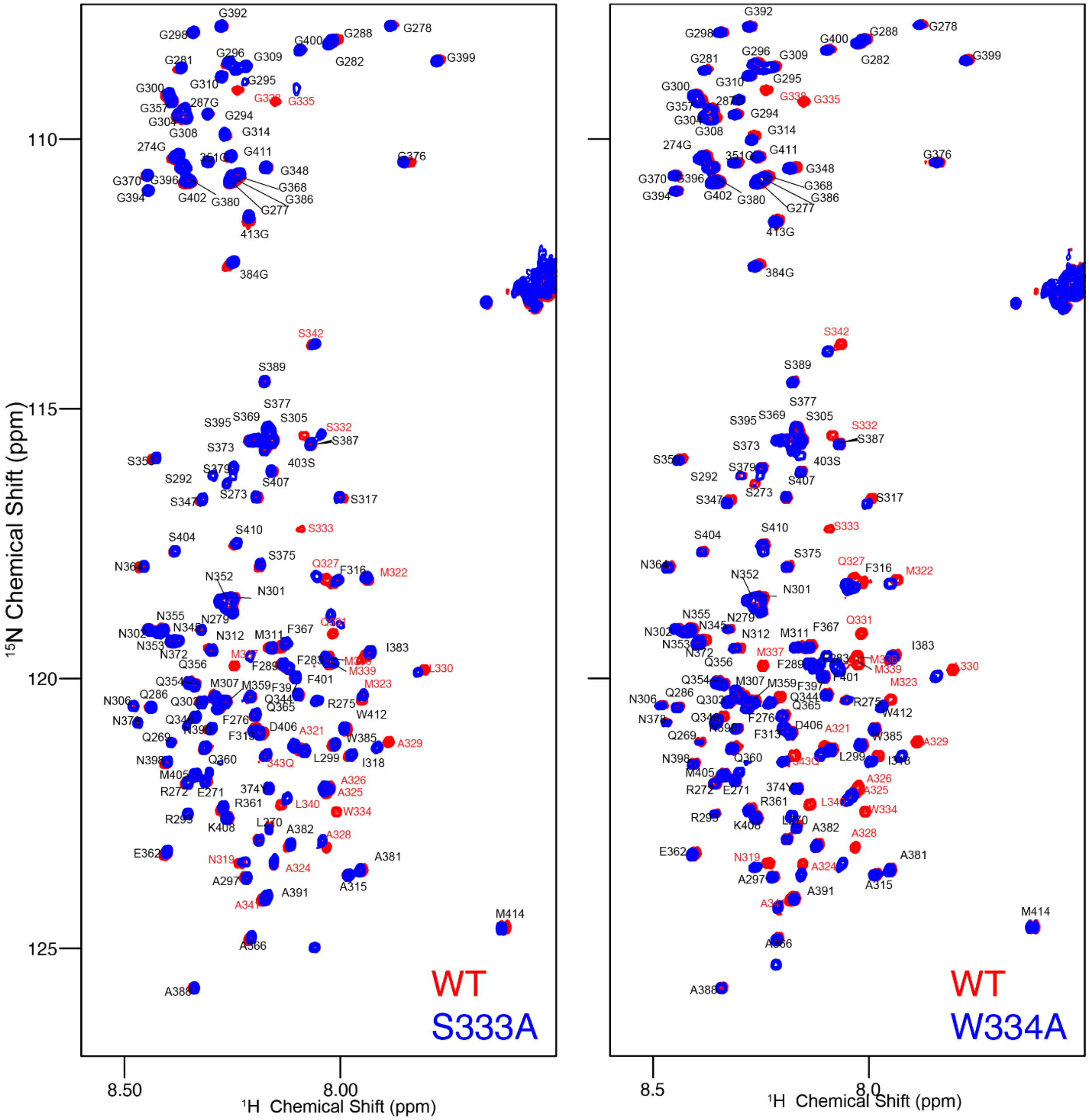

Supplementary Figure 2. Comparison of effects of example alanine variants on NMR spectra of monomeric TDP-43 CTD. S333A (left) shows spectral changes near the mutation site as expected for a covalent modification but shows little change to the CR – for example helical M322, A324, A325, A326 that are distant from the site of mutation show little change in helicity. W334A (right), however, shows changes at these positions far from the mutation site, consistent with reduction in helical population.

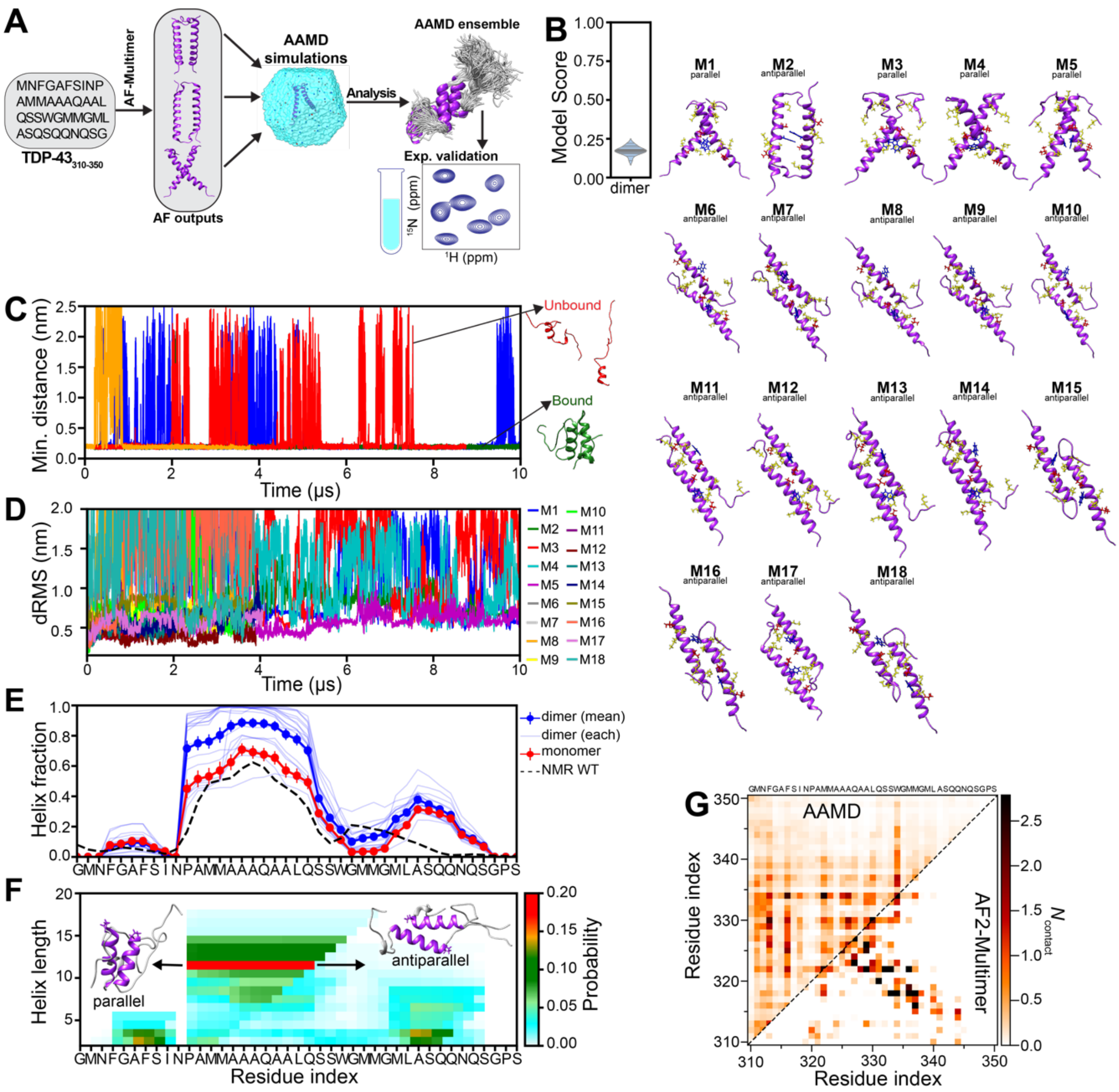

Supplementary Figure 3. **Characterization of TDP-43$_{310\text{–}350}$ dimers from two-chain all-atom molecular dynamics (AAMD) simulations**. **A.** Schematics of simulation setup: Dimer structures of TDP-43$_{310\text{–}350}$ were predicted using AlphaFold2-Multimer tool, then fed to run all-atom molecular dynamics (AAMD) simulations. These simulations were combined to form an ensemble (total runtime ~100 μs) of TDP-43$_{310\text{–}350}$ dimers for analysis and comparison to the experimental data. **B**. The model confidence score and initial structures of AF2-Multimer predicted dimers used for AAMD simulations **C.** Time evolution of minimum distance between the TDP-43$_{310\text{–}350}$ chains in two-chain simulations show their dynamic nature with dissociation and re-association of helices **D**. Distance root-mean-square (dRMS) of all heavy atoms (excluding hydrogen atoms) for residues from 320 to 341 as a function of time with respect to initial conformations are computed from two-chain all-atom simulations. Time evolution of dRMS is shown for 18 independent trajectories. **E.** Per-residue helix fraction (mean ± SEM from 18 replicas) of TDP-43$_{310\text{-}350}$ chains, calculated from TDP-43$_{310\text{-}350}$ dimer (fractions from 18 individual simulations shown as lighter color) and monomer (mean ± SEM from 5 replicas) simulations, compared with helix fractions from NMR-derived ensembles of TDP-43 CTD. **F**. Helix position and length map for TDP-43$_{310\text{–}350}$ (computed averaging over 18 trajectories of TDP-43$_{310\text{–}350}$ dimers) show the highest probability of helices spanning from aa. 320 to 331. The representative snapshots corresponding to the most populated helix length show both parallel and antiparallel helix binding orientations. **G**. Pairwise intermolecular contact map as a function of residue

index from two-chain AAMD simulations is compared to initial contacts in dimer structures from AF2-Multimer. Source data are provided as a Source Data file.

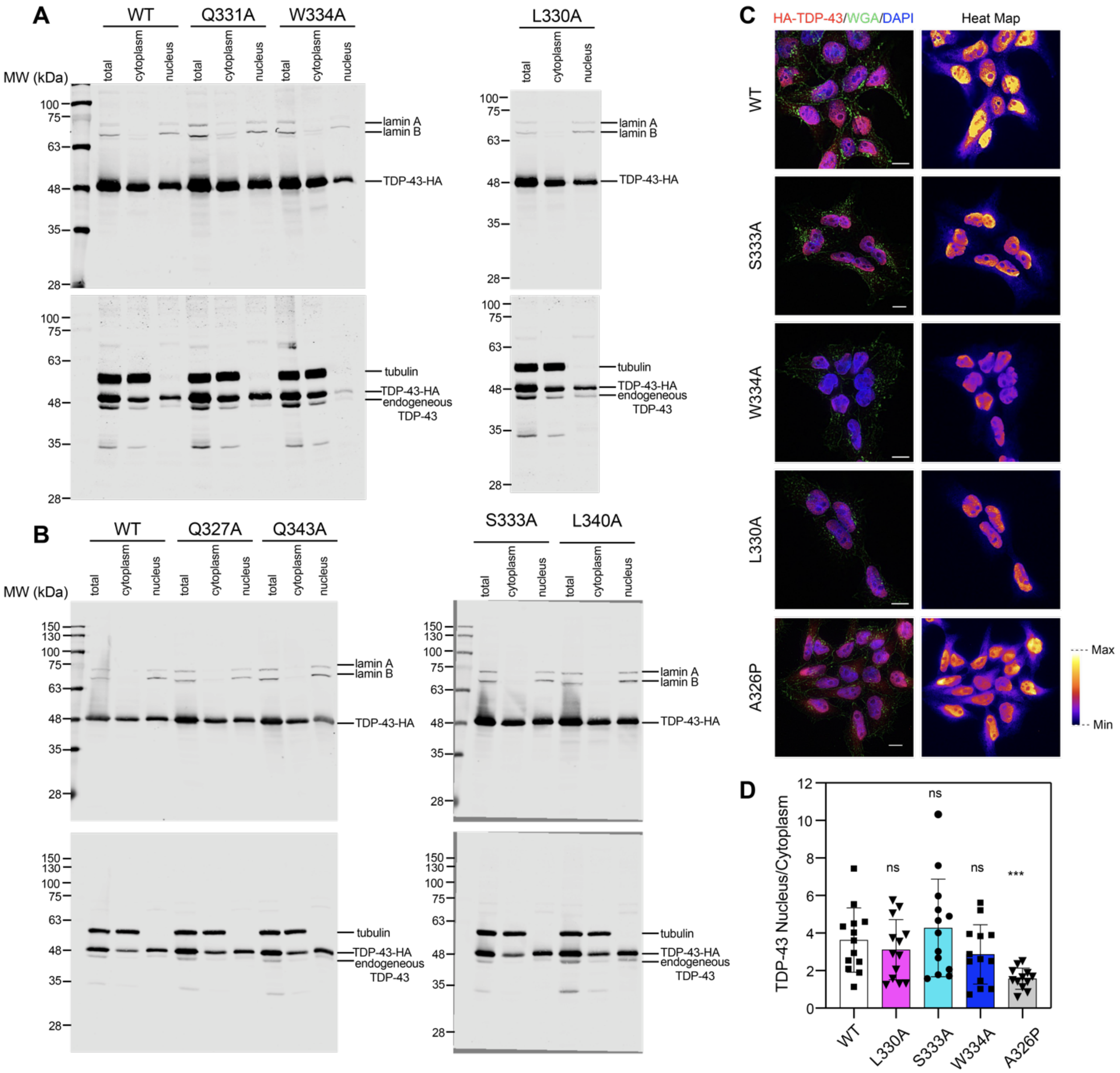

Supplementary Figure 4. TDP-43 CR helix assembly affects TDP-43 nuclear retention. A-B. Representative immunoblot of total, cytoplasmic and nuclear fractions of $HEK293^{HA\text{-}TDP\text{-}43}$ cells from the individual experiments that were performed together. Lamin A/C and tubulin were used as nuclear and cytoplasmic controls, respectively, ensuring proper separation and integrity of each subcellular compartment. Total TDP-43 protein levels were assessed by probing the membrane with an antibody against TDP-43 (bottom panel), while (HA)-tagged TDP-43 was specifically detected using an anti-HA antibody. **C**. Quantification of TDP-43 nuclear to cytoplasmic ratio using immunofluorescence analysis. Stable and isogenic HEK293 cells expressing a single copy of HA-tagged WT or mutant full-length in $HEK293^{HA\text{-}TDP\text{-}43}$ probed with anti-HA antibody and stained with DAPI and wheat germ agglutinin (WGA) to define the nucleus and the cytoplasmic area, respectively (top: immunofluorescence, bottom: pseudo-color look-up table). The intensity histogram for each image was independently maximized across the full range. Scale bar, 10 μm. **D**. Nuclear and cytoplasmic HA-TDP-43 intensity was calculated using standard protocols in ImageJ. Graph shows the mean ± SD of HA-TDP-43 nuclear/cytoplasmic ratio using from ≥ six individual replicates, where >300 individual cells were quantified in total. Statistical analysis was assessed using a two-sided Mann-Whitney U test, ***p = 0.0003. Source data are provided as a Source Data file.

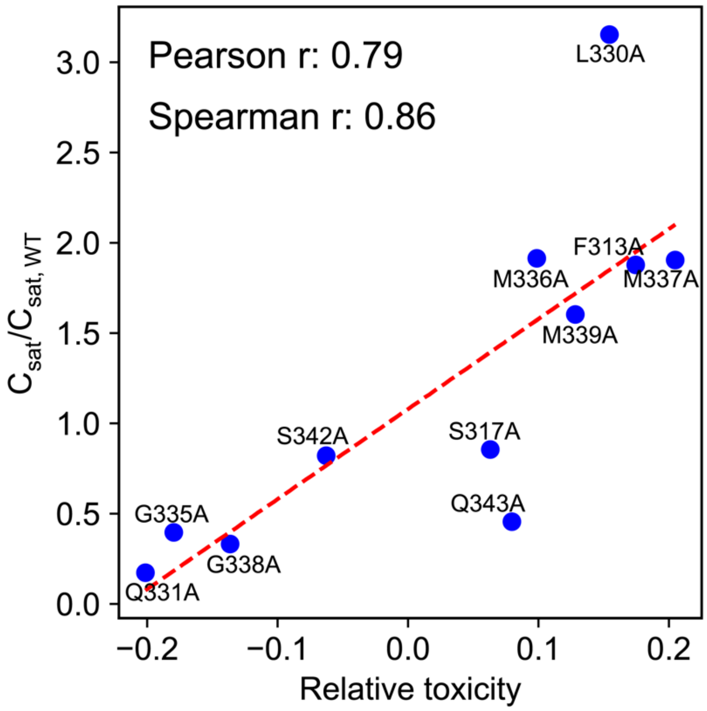

Supplementary Figure 5. The relative toxicity of single alanine substitution variants at non-alanine positions within the CR and adjacent residues in yeast cells[1] shows a strong correlation (Pearson correlation coefficient, (r = 0.79) and Spearman's rank correlation coefficient, (r = 0.86)) with the C_{sat}, normalized by the C_{sat} of the WT CTD, from single alanine substitution variants at the same positions. Source data are provided as a Source Data file.

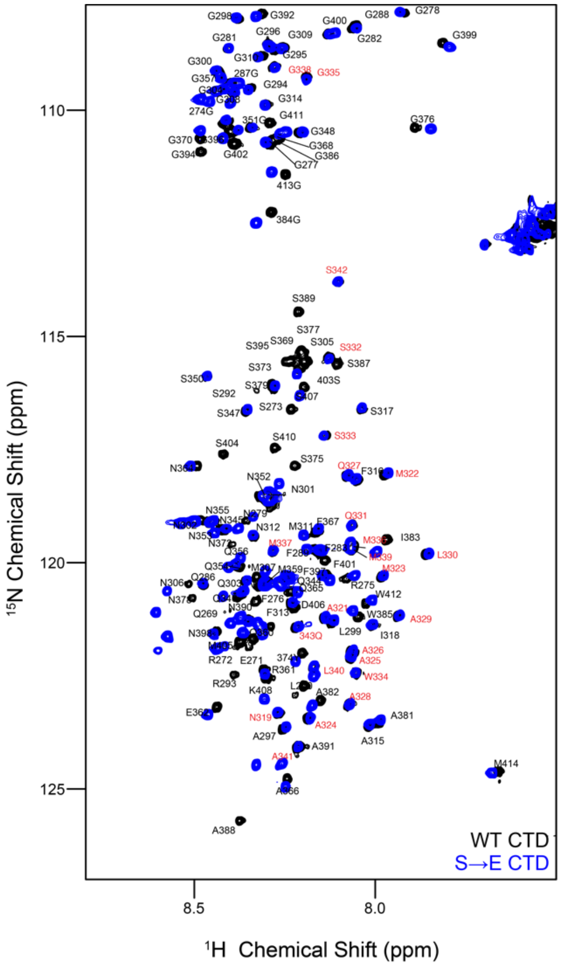

Supplementary Figure 6. Mutations in the flanking region dramatically enhance solubility. while preserving CR structure. A comparison of the ^{1}H-^{15}N HSQC of 20μM WT CTD (black) and 20 μM S→E CTD (blue) in 20 mM MES (pH 6.1) at 25ºC. The spectra are highly similar for the CR (red labels) where the resonances overlay nearly perfectly, suggesting no change in the structure of the CR is induced by the S→E substitutions.

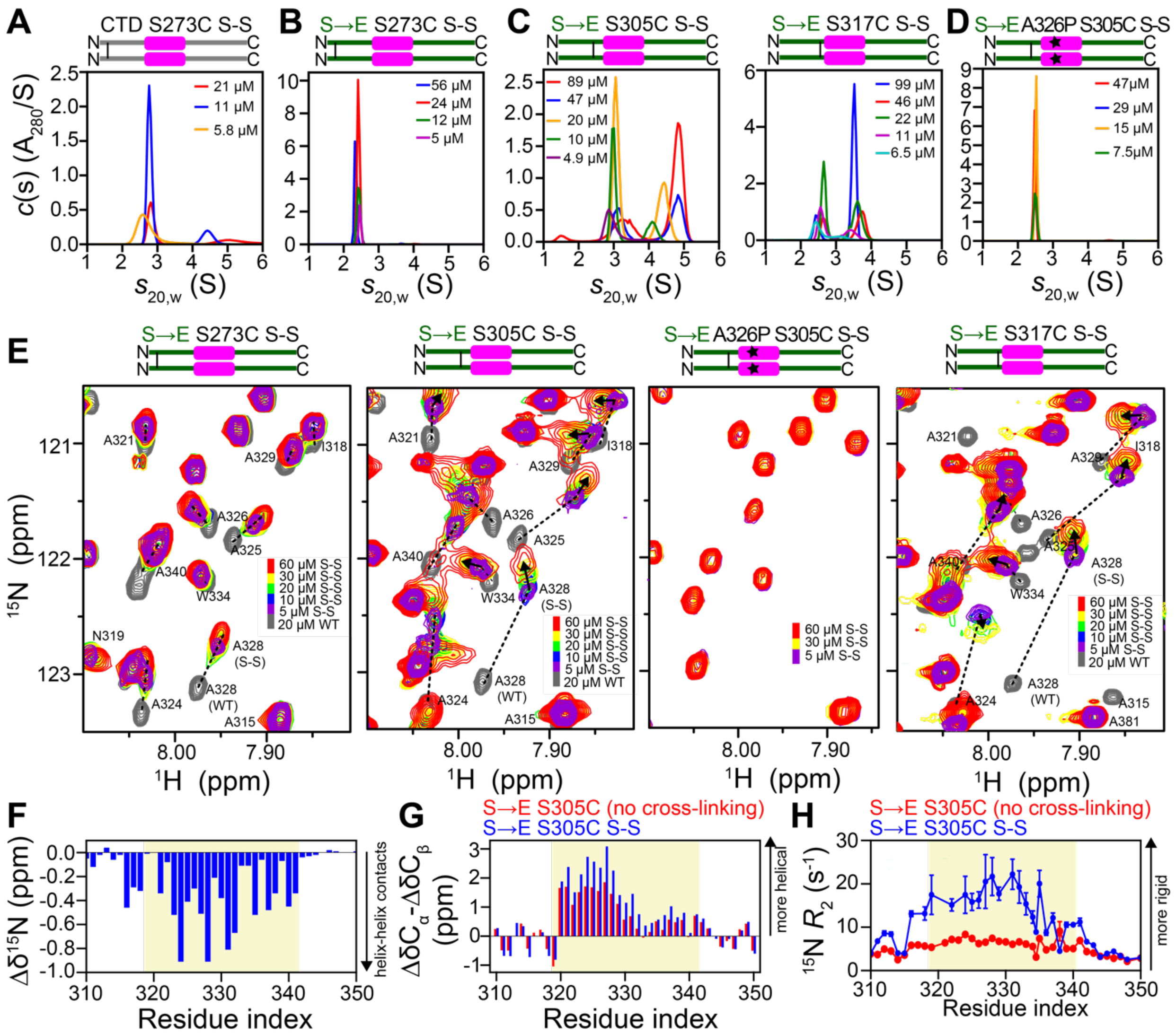

Supplementary Figure 7. AUC and NMR Characterization of Designed CTD variants A. Sedimentation velocity analytical ultracentrifugation (SV-AUC) of CTD cysteine cross-linked dimers at S273C as a function of protein concentration. Data were collected in 12 mm cells, except for at 21 μM, where 3 mm pathlength cells were used. **B**. SV-AUC of soluble CTD variant cross-linked at S273C (S→E S273C) as a function of protein concentration. Data were collected in 12 mm cells, except for at 56 μM, where 3 mm pathlength cells were used. **C**. SV-AUC of soluble CTD variant cross-linked closer to CR, at S305 C (S→E S305C) and at S317C (S→E S317C) as a function of protein concentration. **D**. SV-AUC of S→E S305C with helix-disrupting A326P variant as a function of protein concentration. SV-AUC data for S→E S305C and S317C variants show the presence of higher-order assemblies (tetramer (~3.8 S) and octamer (~4.5 S)) along with dimers (~2.8 S), whereas only dimers are observed for the helix-disrupting S→E S305C A326P variant. Data were collected in 12 mm cells, except for S→E S305C at 47 and 89 μM, S→E S317C at 46 and 99 μM, and S→E S305C A326P at 29 and 47 μM, where 3 mm pathlength cells were used. **E**. NMR chemical shift analysis of S→E S273C, S→E S305C and S→E S317C as a function of increasing protein concentration exhibits distinct chemical shift changes distinct from dimer formation, suggesting formation of higher order multimers with additional new contacts. Helix-disrupting mutant, A326P on S→E S305C dimer does not show chemical shift deviations up to 60 μM protein concentrations, suggesting the multimerization is primarily mediated by helix-helix interaction. **F**. Quantification of 15N Δδ values between dimeric (cross-linked, oxidized) and monomeric (reduced with 1mM DTT) S→E S305C variants. The concentration of cross-linked S→E S305C is 30 μM, while monomeric S→E S305C (reduced) is 60 μM. Measurements performed in 20 mM MES buffer, pH 6.1 at 47ºC. **G**. Experimental NMR secondary chemical

shifts ($\Delta\delta C\alpha$-$\Delta\delta C\beta$) of dimeric (cross-linked, oxidized) and monomeric (reduced) S→E S305C variants show the increase in helicity with cross-linking for entire CR residues. **H**. ^{15}N R_2 parameters (mean ± SD) for cross-linked (oxidized) and monomeric (reduced) S→E S305C variants suggest slowed reorientation motions for the 315–343 region upon cross-linking. Source data are provided as a Source Data file.

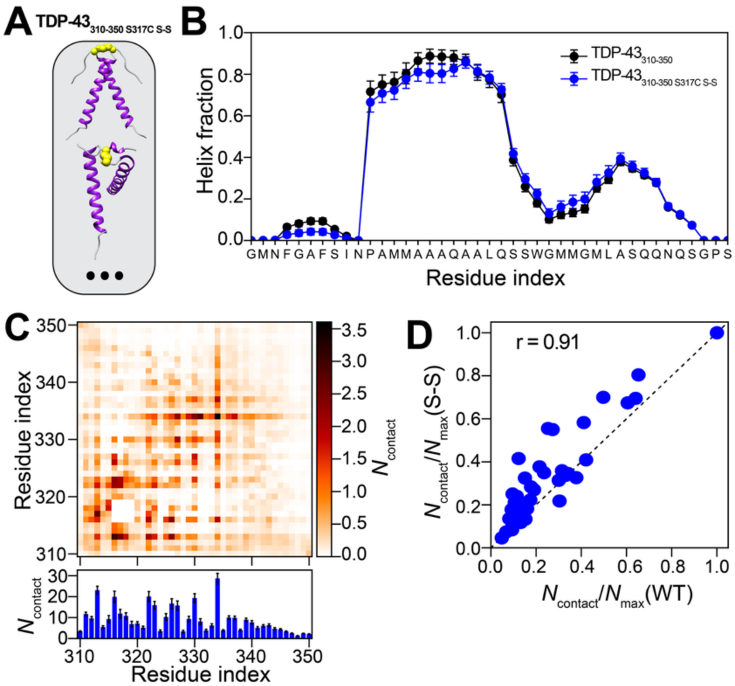

Supplementary Figure 8. **Cross-linking near CR is expected to result in similar helicity and helix-helix contacts as in WT CTD dimers. A**. TDP-43310–350 cross-linked dimers are modelled from AF2-Multimer predicted dimer models by adding a disulfide bond (S-S) at S317C. Then, 8 different starting conformations of the cross-linked dimers, TDP-43310–350_S317C_S-S are fed to run AAMD simulations (3 independent runs for each model, 24 runs in total), resulting in a well-converged ensemble (total runtime ~45 μs). **B**. Per–residue helix fraction of TDP-43310–350 chains calculated from TDP-43310–350 dimer (WT) (mean ± SEM from 18 independent runs) and TDP-43310–350_S317C_S-S cross-linked dimer (mean ± SEM from 18 independent runs) simulations. TDP-43 cross-linked dimer shows helix content similar to those observed in the wild-type. **C**. A two-dimensional pairwise intermolecular contact map as function of residue index (top) and total number of intermolecular contacts per residue position ($N_{contact}$) (bottom) is computed from of the simulated cross-linked dimer ensemble. Contacts averaged over 24 trajectories; 1D map shows mean ± SEM **D**. Per-residue intermolecular contacts (normalized by maximum contact in each case) calculated from TDP-43310–350 (WT) and cross-linked (S-S) dimer simulations show a strong correlation (Pearson correlation coefficient, r = 0.91). Source data are provided as a Source Data file.

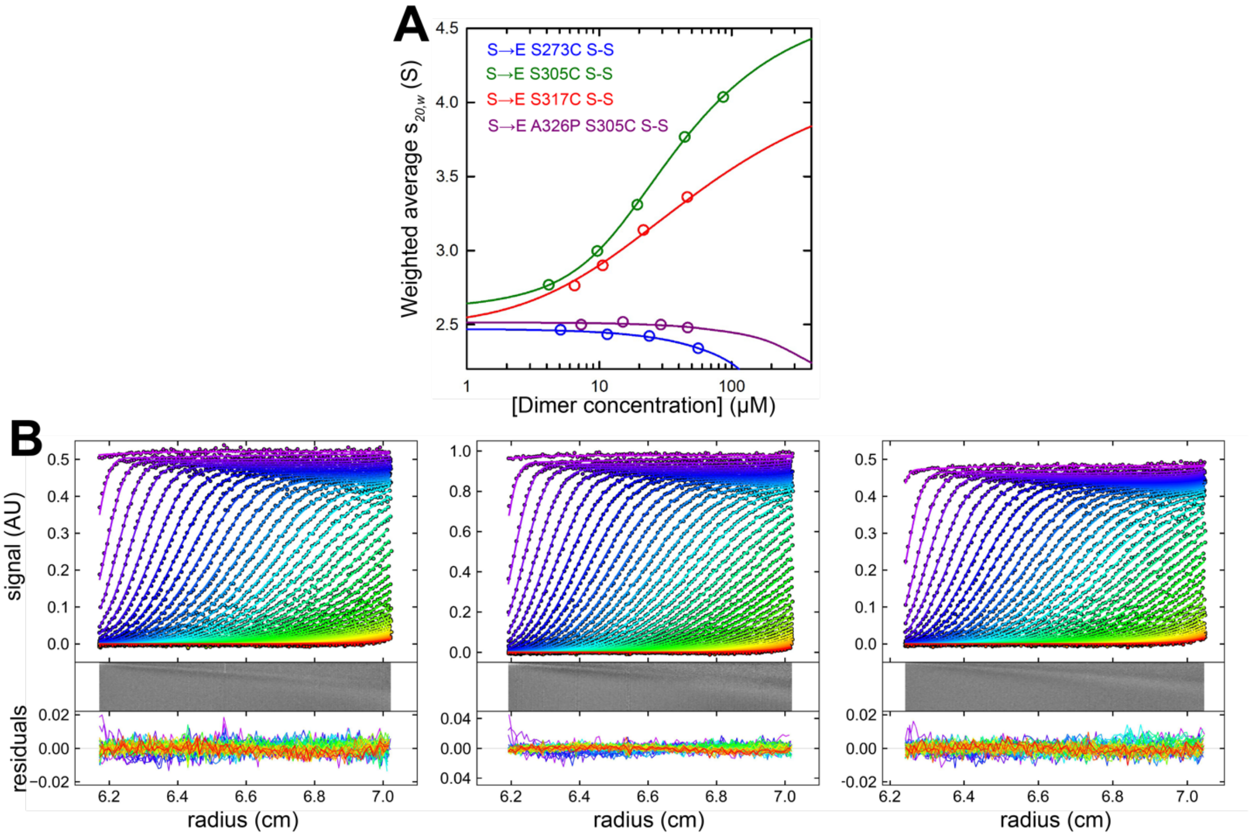

Supplementary Figure 9. **AUC Characterization of Designed CTD variants A**. Weighted average sedimentation coefficient isotherms as a function of protein concentration based on the sedimentation velocity c(s) distributions. S→E S273C (blue) and S→E S305C A326P (purple) do not self-associate and presented as dimers at all concentrations studied. For these variants, a linear decrease (best-fit function) used to describe the concentration dependence of the sedimentation coefficient accounts for non-ideality. Because the concentration is presented on a logarithmic scale in concentration, the fit appears non-linear. S→E S317C (red) were modeled in terms of a dimer – tetramer reversible self-association to obtain a dimer – tetramer K_d of 45 μM (in dimer concentration units). S→E S305C (green) were modeled in terms of a dimer – tetramer – octamer reversible self-association to obtain a dimer – tetramer K_d of 58 μM and a tetramer – octamer K_d of 4.7 μM, indicative of cooperative self-assembly. **B.** Absorbance sedimentation data collected for S→E S317C at 280 nm and (left) 47 μM (3 mm pathlength cell), (center) 22 μM (12 mm pathlength cell), and (right) 11 μM (12 mm pathlength cell) were analyzed globally in terms of a dimer – tetramer self-association using Lamm equation modeling, along with trace amounts of an aggregate. The analysis, carried out in SEDPHAT[2], returns a dimer – tetramer K_d of 46 μM (68% confidence interval of 42 – 51 μM). Data were plotted in GUSSI[3] and for clarity only every third scan and every third experimental data point are shown. Best-fits are represented by a solid line through the experimental points. A bitmap representation of the residuals, together with the combined residuals, are shown below each plot. Source data are provided as a Source Data file.

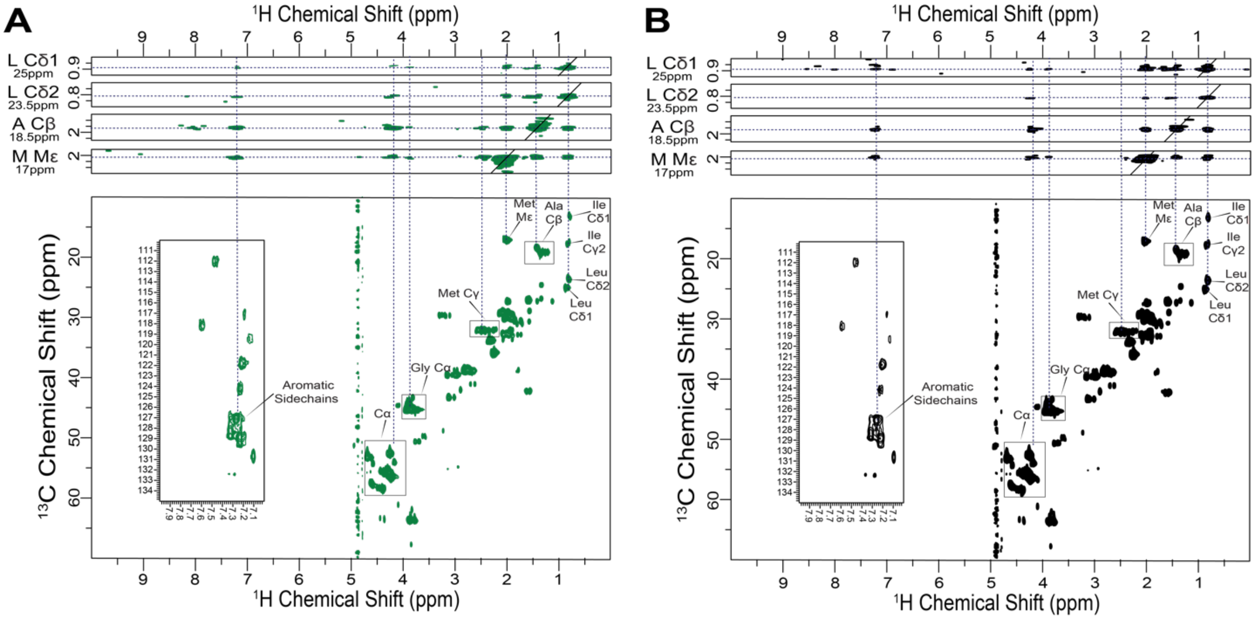

Supplementary Figure 10. Intermolecular NOEs for cross-linked variants show key contacts for TDP-43 CR assembly. ^{13}C filtered-edited NOE-HSQC strips of S→E S273C (**A**) and S305C (**B**) cross-linked dimers. Cross-linked dimers were formed 1:1 mixture of ^{13}C/^{15}N labeled and unlabeled monomers in the presence of 1 mM copper(**II**) phenanthroline catalyst. Spectra were recorded at 47 ºC using a dimer concentration of 70 µM in 20 mM MES pH 6.1 with 10% D_2O.

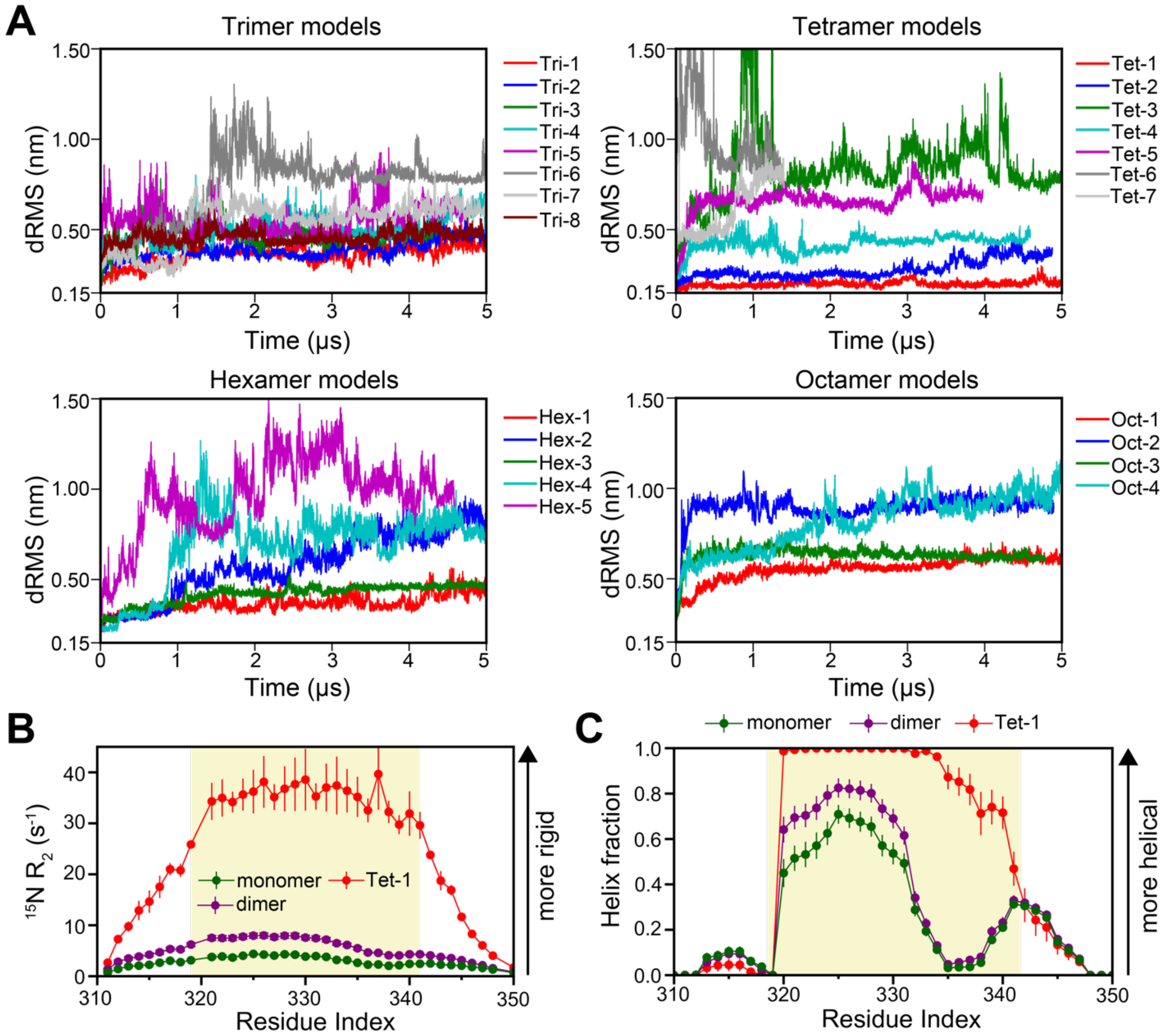

Supplementary Figure 11. Structural models of the multimeric helical assembly of TDP-43 CTD. A. Time evolution of distance root-mean-square (dRMS) from the AAMD simulations of AF2-Multimer predicted multimer (trimer to octamer) models (~4-5 μs each). dRMS of all heavy atoms (excluding hydrogen atoms) for residues from 320 to 341 with respect to initial conformations are shown. **B.** ^{15}N R_2 of Tet-1 from AAMD simulations (single run, 5 μs) (mean ± SEM using block averaging over 5 time blocks) suggests slower motions compared to that from monomeric (mean ± SEM from 5 independent runs) and dimeric (mean ± SEM from 18 independent runs) AAMD ensembles, consistent with results from Supplementary Figure 7H. **C.** Per-residue α-helical fraction from Tet-1 AAMD simulations (single run, 5 μs) (mean ± SEM using block averaging over 5 time blocks) show increase in helicity in the main helical region (aa: 320-331) and further helix extension in the adjacent 332–343 region with helix-helix assembly compared to that from monomeric (mean ± SEM from 5 independent runs) and dimeric (mean ± SEM from 18 independent runs) AAMD ensembles. Source data are provided as a Source Data file.

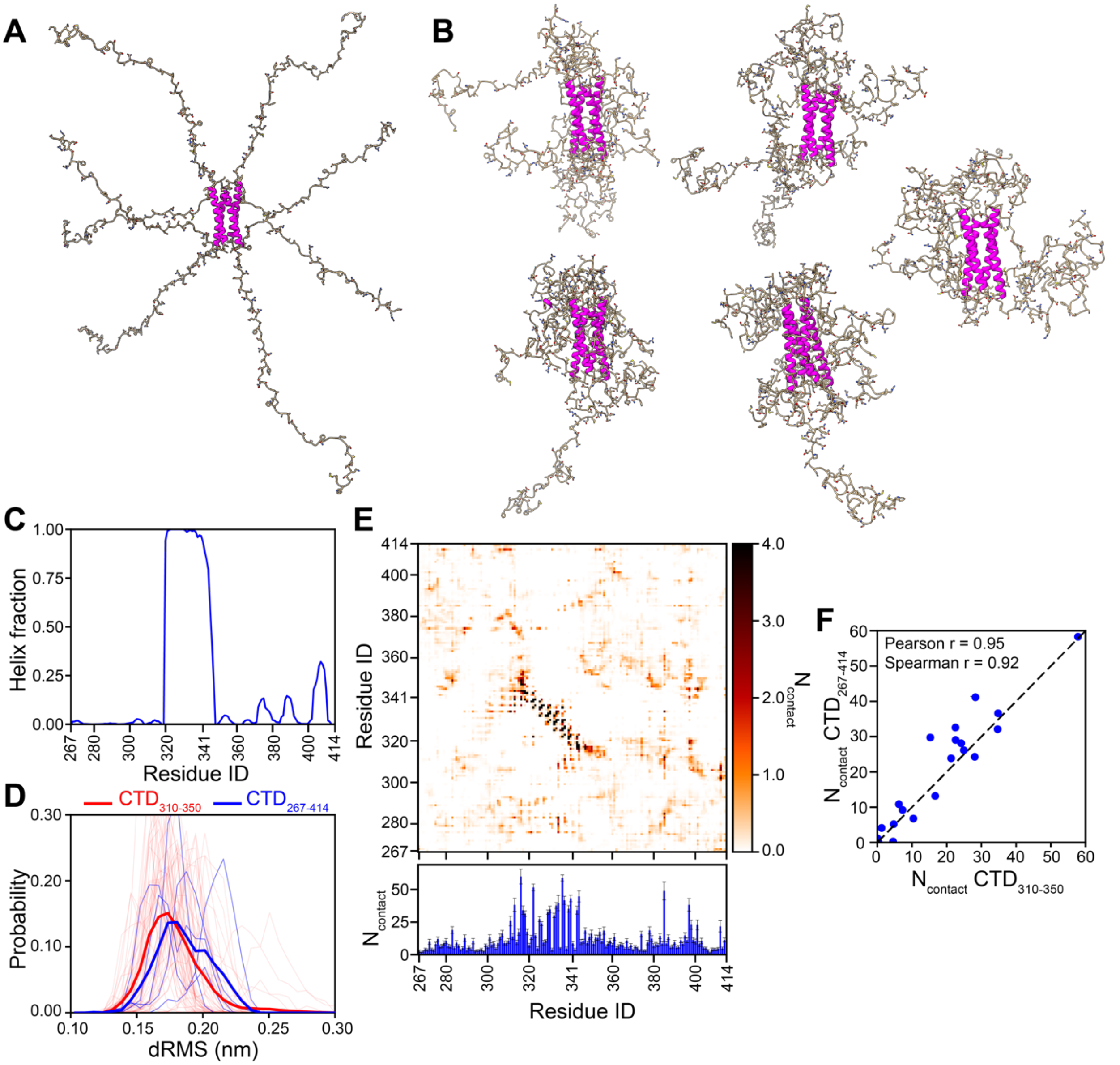

Supplementary Figure 12. Analysis of CTD full-length tetramer simulations. A. Full-length CTD (aa: 267-414) tetramer model generated using MODELLER[4], using Tet-1 structure for CR residues (aa: 319-341). **B**. Five representative CTD tetramer structures used as starting conformations for all-atom MD simulations. These structures were obtained from CG simulations of the model shown in (A) and subsequently back-mapped to atomistic resolution using MODELLER[4]. **C.** Per-residue α-helical fraction computed from CTD full-length tetramer AAMD simulations, averaged across five trajectories, highlighting the stability of helicity within the CR region (residues 320–341). **D**. dRMS distributions from AAMD simulations of Tet-1 model ($CTD_{310-350}$) (50 runs with 100 ns each) compared to CTD full-length ($CTD_{267-414}$) tetramer (5 independent runs with 300 ns each). dRMS distribution of all heavy atoms (excluding hydrogen atoms) for residues from 320 to 341 as a function of time with respect to initial conformations are shown. dRMS for CR tetramer of $CTD_{310-350}$ and $CTD_{267-414}$ shown in red and blue colors, respectively. The lighter colors represent the dRMS distributions for independent runs, while the darker colors represent the average dRMS **E**. (top) Pairwise intermolecular contact maps of CTD full-length tetramer simulations (bottom) Total number of contacts per residue position ($N_{contact}$) derived through summation of all pairwise contacts along y-axis based on two dimensional pairwise intermolecular contact maps. Contacts averaged over five trajectories; 1D map shows mean ± SEM. **F.** Per-residue intermolecular contacts within CR (aa:

320-341) calculated from $CTD_{310–350}$ and $CTD_{267-414}$ tetramer simulations show a strong correlation (Pearson correlation coefficient, r = 0.95, Spearman correlation coefficient, r = 0.92). Source data are provided as a Source Data file.

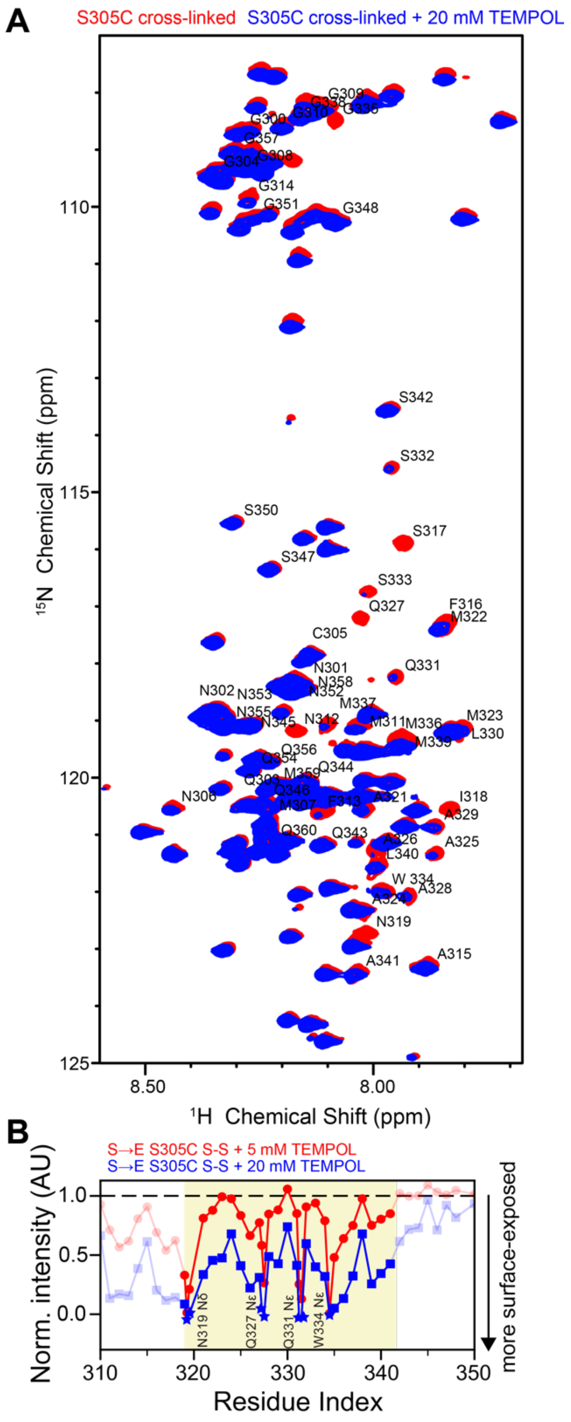

Supplementary Figure 13. Solvent PRE analysis of TDP-43 S→E S305C cross-linked dimer variant. **A**. ^{1}H-^{15}N HSQC of 30 μM S→E S305C cross-linked dimer without (red) and with 20 mM TEMPOL (blue) at 47ºC, 850 MHz **B**. Normalized intensity ratio at 5 and 20 mM TEMPOL concentrations are shown. Peak intensities were normalized based on the sample without TEMPOL. Source data are provided as a Source Data file.

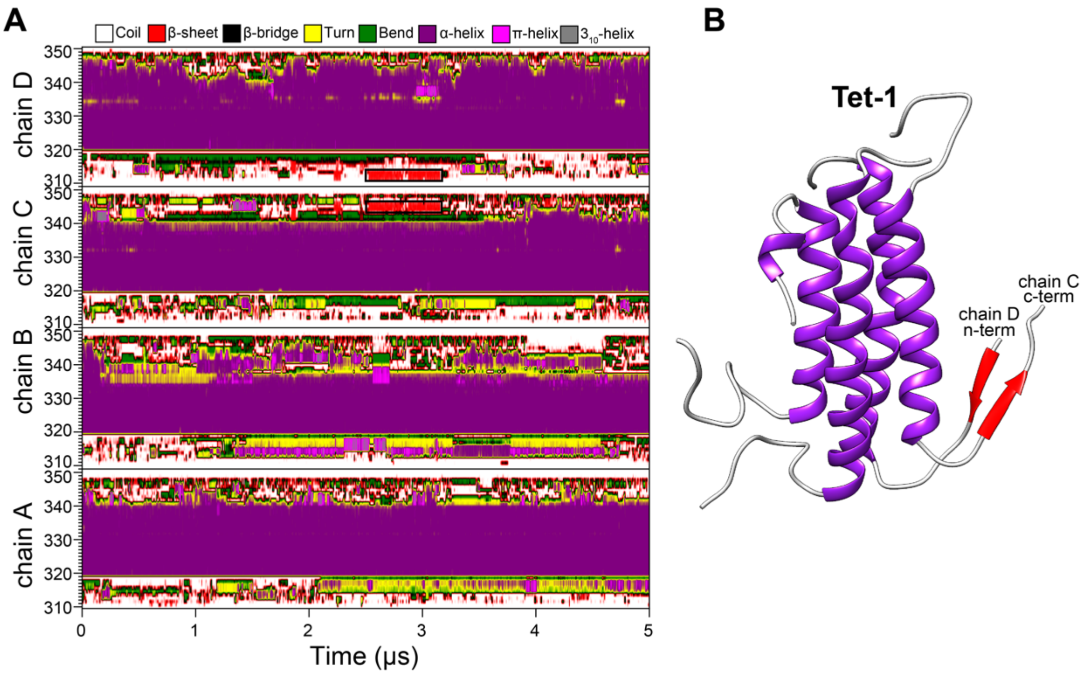

Supplementary Figure 14. Secondary structure analysis from AAMD simulations of Tet-1. Secondary structure change as a function of time in Tet-1 AAMD simulation trajectory (single run, 5 μs) shows the formation of intermolecular β-sheet-structure (highlighted in black boxes). **B**. The snapshot from Tet-1 AAMD simulation that shows the formation of transient, antiparallel β-sheets (red strands) between residues ^{310}GMNF^{313} of chain C and residues ^{344}QNQ^{346} of chain D. Source data are provided as a Source Data file.

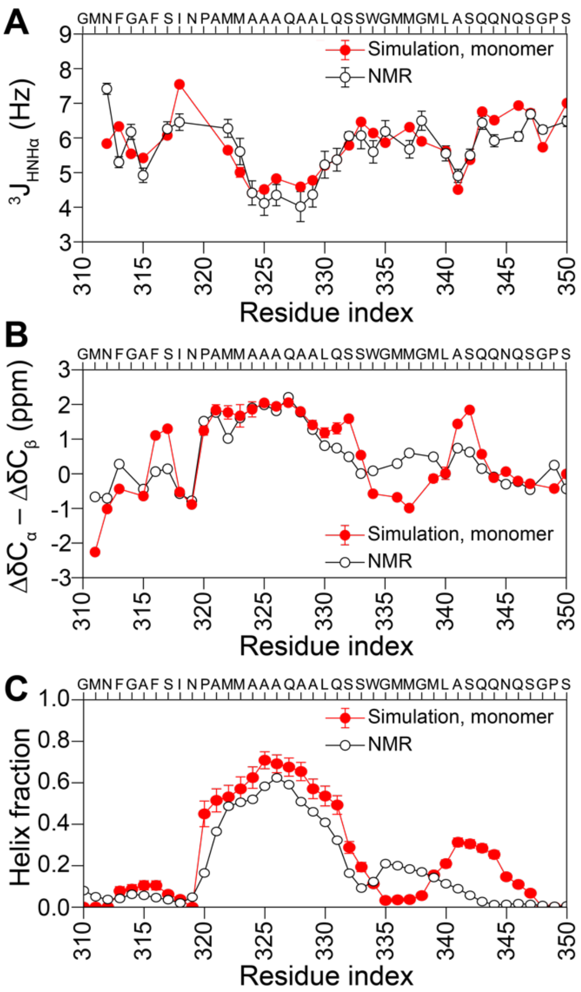

Supplementary Figure 15. **Assessing the suitability of Amber03ws force field for TDP-43$_{310\text{-}350}$. A**. Comparison of $^3J_{HNH\alpha}$ scalar coupling constants computed from unbiased single-chain simulations of TDP-43$_{310–350}$ (aggregate time = 45 μs) using MDTraj, following the Bax2007 model[5] with the experimental $^3J_{HNH\alpha}$ scalar coupling constants (BMRB 26823 [https://dx.doi.org/10.13018/BMR26823])[6]. Standard errors are calculated over 5 independent trajectories. **B**. Comparison of per-residue secondary chemical shift differences ($\Delta\delta C_\alpha$–$\Delta\delta C_\beta$ shifts) computed from the simulation trajectory using SPARTA+ algorithm[7] with the experimental secondary chemical shifts (BMRB 26823)[6]. Standard errors are calculated over 5 independent trajectories **C**. Comparison of per-residue helical fractions from unbiased single-chain simulations using DSSP algorithm[8] with NMR-derived helix fractions based on experimental chemical shifts (BMRB 26823)[6] using δ2D[9]. Standard errors are calculated using 5 independent trajectories. Source data are provided as a Source Data file.

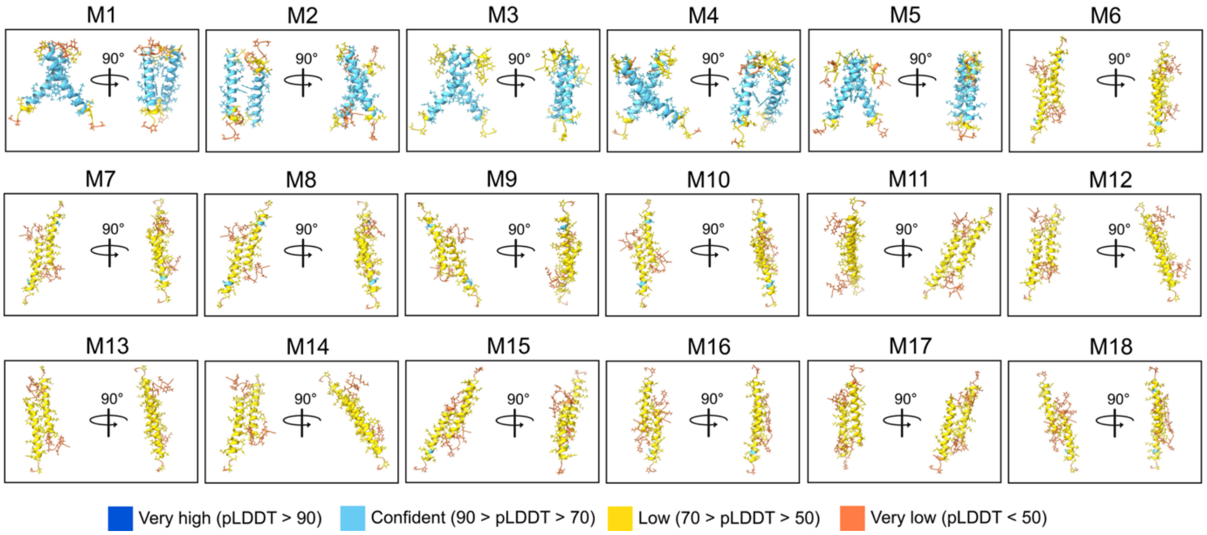

Supplementary Figure 16. 18 Dimer structure models used in the AAMD simulations, colored according to per-residue pLDDT scores. The first 5 models were predicted with AF2-Multimer version 2.2.0, and the remaining 13 with AF2-Multimer version 2.3.0. Source data are provided as a Source Data file.

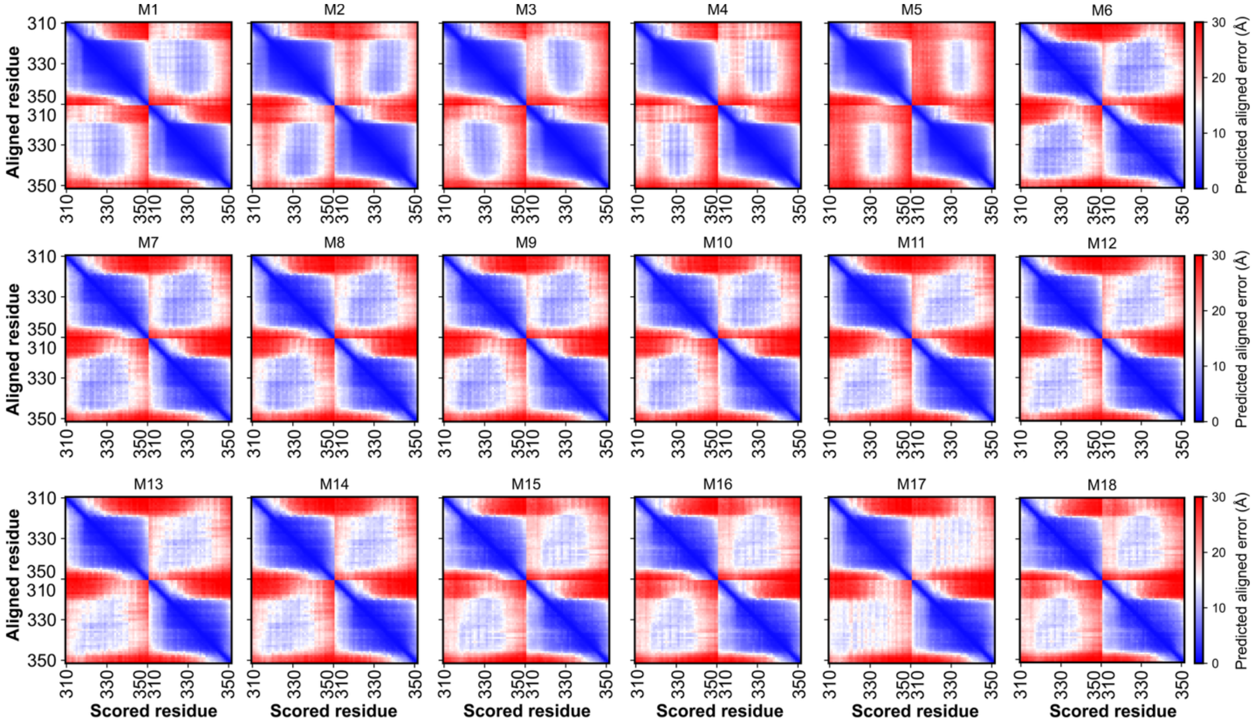

Supplementary Figure 17. Predicted Aligned Error (PAE) maps of 18 dimer structure models used in the AAMD simulations. Source data are provided as a Source Data file.

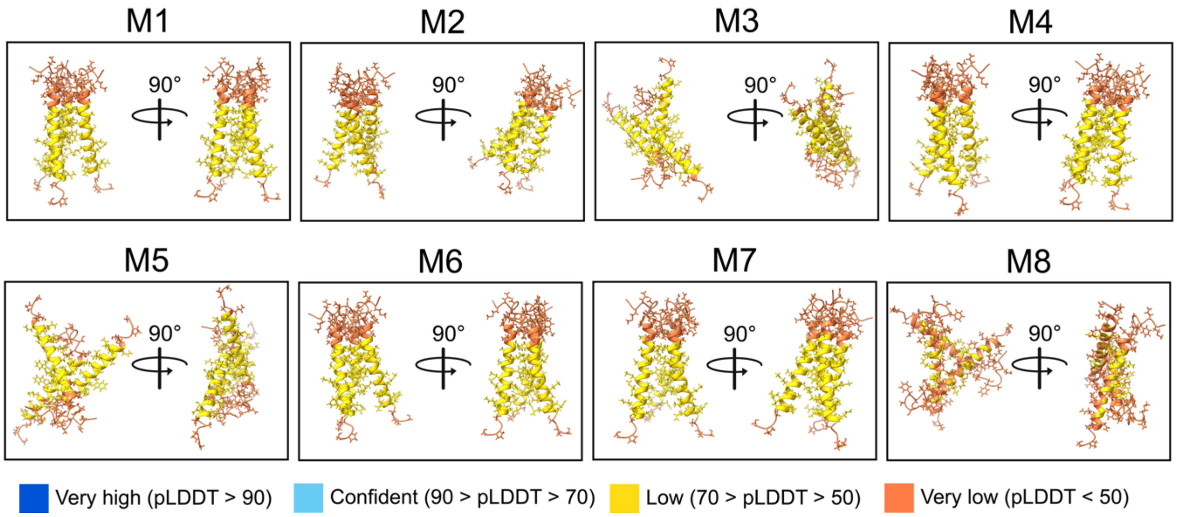

Supplementary Figure 18. 8 Trimer structure models used in the AAMD simulations, colored according to per-residue pLDDT scores. The models were predicted with AF2-Multimer version 2.3.0. Source data are provided as a Source Data file.

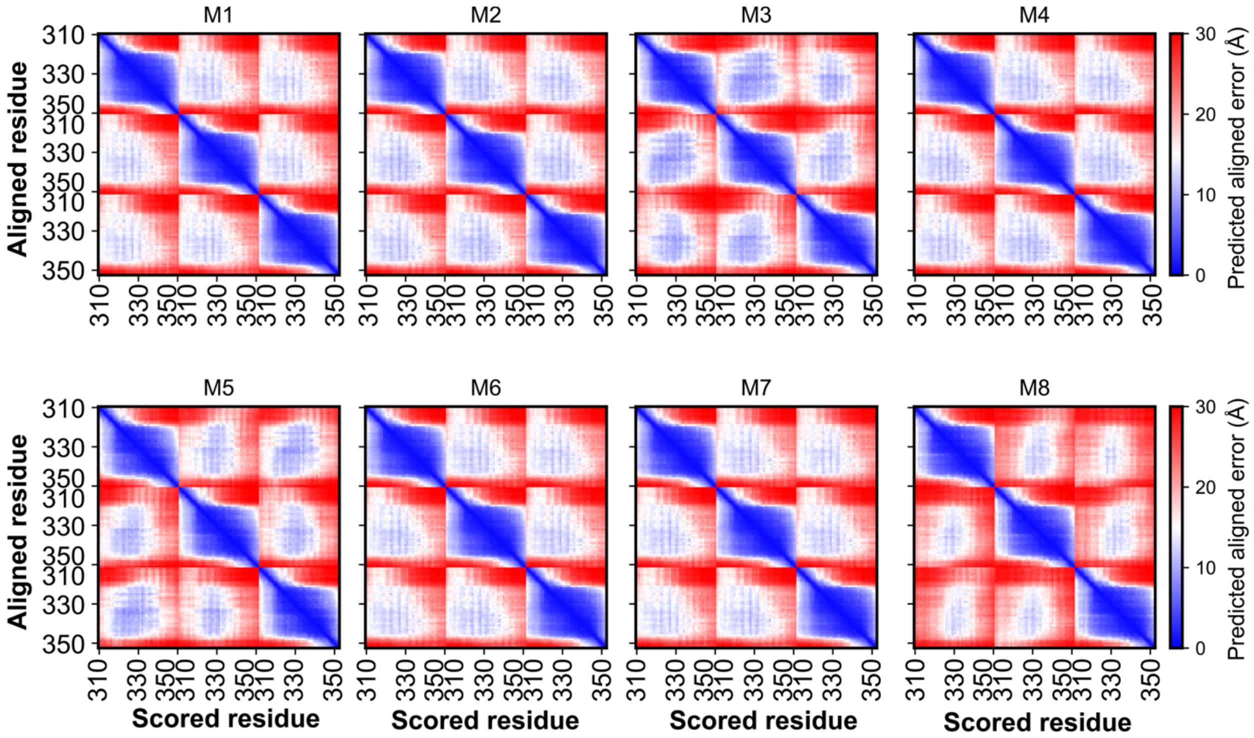

Supplementary Figure 19. PAE maps of 8 trimer structure models used in the AAMD simulations. Source data are provided as a Source Data file.

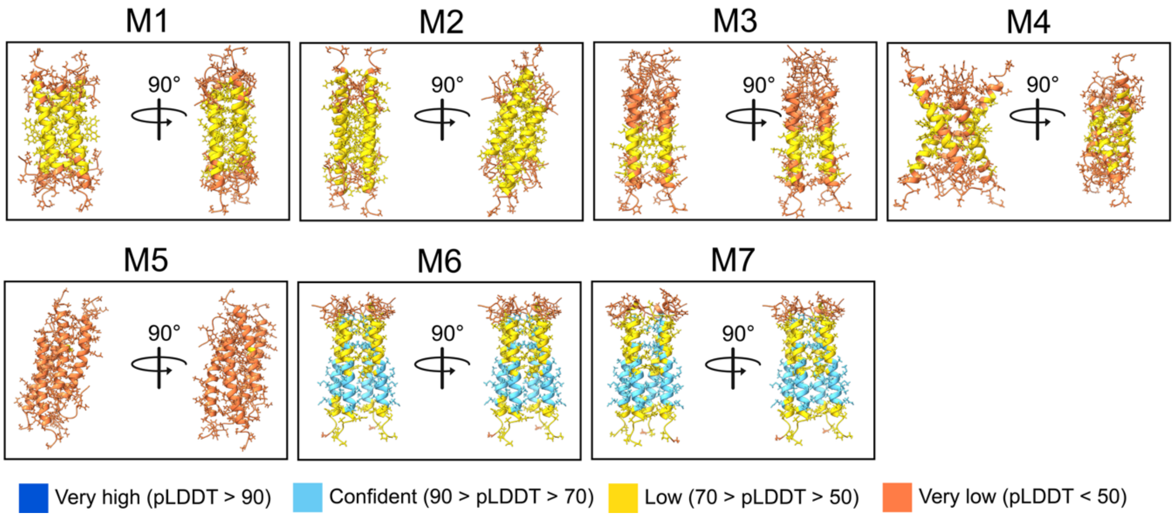

Supplementary Figure 20. 7 Tetramer structure models used in the AAMD simulations, colored according to per-residue pLDDT scores. The first 5 models were predicted with AF2-Multimer version 2.3.0 (v2.3.0), and the remaining 2 with AF2-Multimer version 2.2.0 (v2.2.0). Source data are provided as a Source Data file.

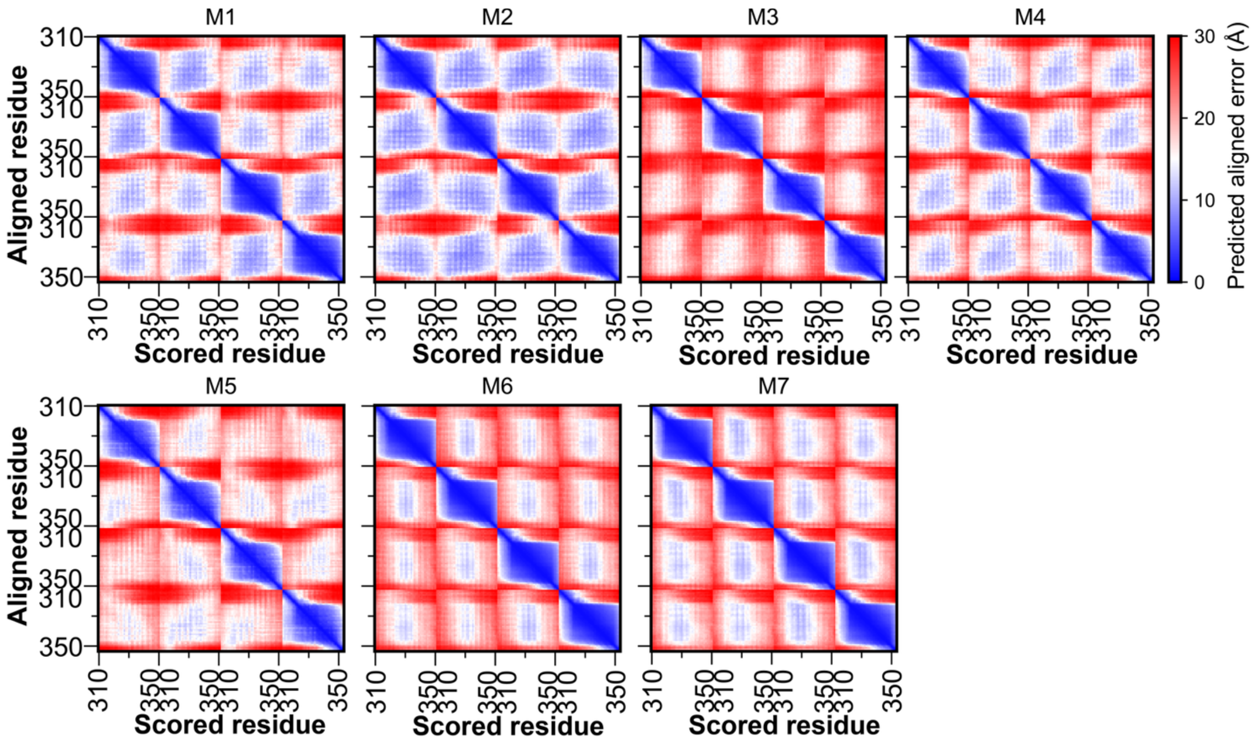

Supplementary Figure 21. PAE maps of 7 tetramer structure models used in the AAMD simulations. Source data are provided as a Source Data file.

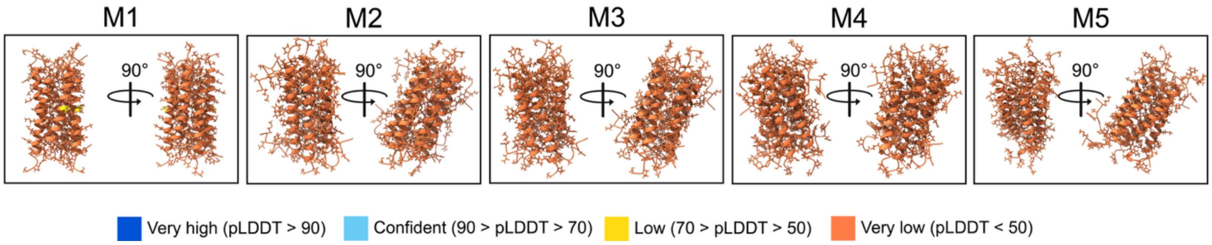

Supplementary Figure 22. 5 Hexamer structure models used in the AAMD simulations, colored according to per-residue pLDDT scores. The models were predicted with AF2-Multimer version 2.3.0. Source data are provided as a Source Data file.

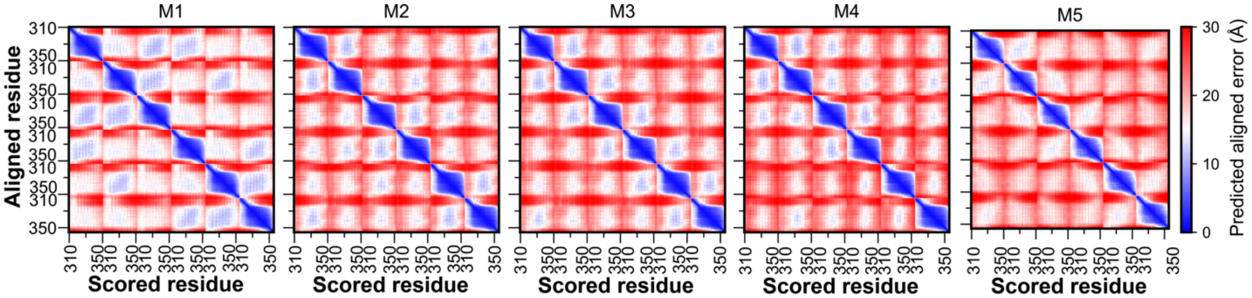

Supplementary Figure 23. PAE maps of 5 hexamer structure models used in the AAMD simulations. Source data are provided as a Source Data file.

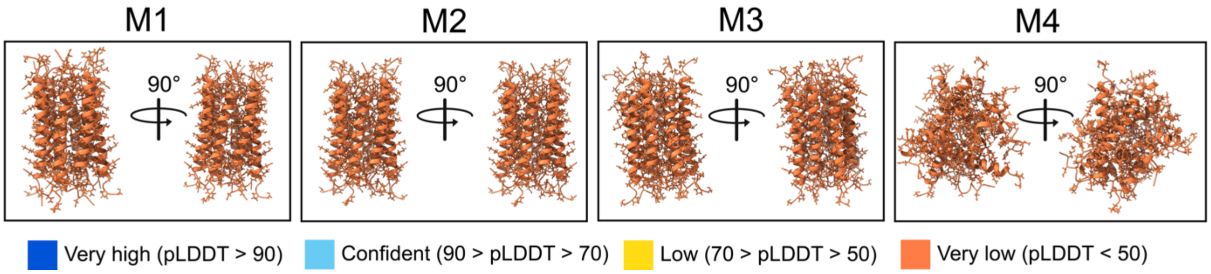

Supplementary Figure 24. 4 Octamer structure models used in the AAMD simulations, colored according to per-residue pLDDT scores. The models were predicted with AF2-Multimer version 2.3.0. Source data are provided as a Source Data file.

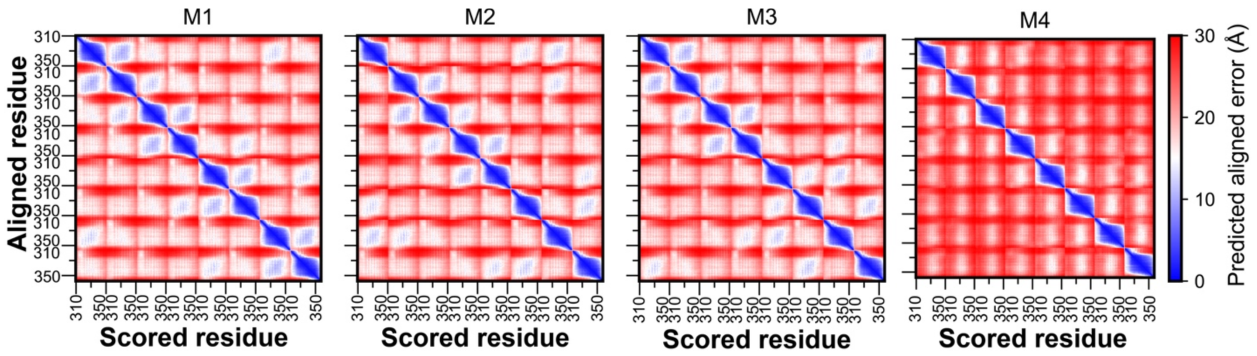

Supplementary Figure 25. PAE maps of 4 octamer structure models used in the AAMD simulations. Source data are provided as a Source Data file.

Supplementary Table 1: Lamm equation modeling SV-AUC data for WT CTD

Model (a)	Best-fit global reduced chi-square	68.3% confidence level critical reduced chi-square (b)	Best-fit sedimentation coefficients (c)	Comments / issues with the best fit parameters for some models
Monomer – dimer – tetramer	0.1657	0.1679	1.54 S, 2.43 S, 3.94 S	
Monomer – tetramer	0.1668	0.1690	1.56 S, 4.38 S	
Monomer – trimer	0.1684	0.1706	1.54 S, 4.07 S	Trimer s-value high
Monomer – dimer – trimer	0.1800	0.1820	1.49 S, 2.67 S, 4.98 S	Trimer s-value too high
Monomer – dimer	0.1865	0.1881	1.45 S, 4.75 S	Dimer s-value too high

(a) All models represent reversible equilibria with mass conservation and include trace contributions (~ 0.01 – 0.015 absorbance units) of a ~10 – 12 S sedimenting aggregate. Global modeling was carried out on absorbance data collected at 107 and 58 μM (see Figures 3A and 3B).

(b) The value of the global reduced chi-square that would lead to a statistically significant (based on one standard deviation or 68.3% confidence level) change based on F-statistics.

(c) Best-fit sedimentation coefficients in order of oligomeric species. In cases where the dimer or trimer sedimentation coefficient was constrained to a range of expected values, the best fit was significantly worse, returning a larger value for the global reduced chi-square.

Supplementary Table 2. Serine positions in TDP-43 CTD simultaneously changed to glutamate in S→E variant series used in NMR experiments, except for positions changed to cysteine for cross-linking.

Mutant	Residues
S→E	S292, S273C, S305, S369, S377, S379, S387, S389, S393, S395, S403, S404, S409, S410

Supplementary Table 3: Oligonucleotides used in these studies.

DNA	Sequence
TDP43_Q327A Fw	5'-GCCATGATGGCTGCCGCCGCTGCAGCACTACAGAGCAGTTGG-3'
TDP43_Q327A Rv	5'-CCAACTGCTCTGTAGTGCTGCAGCGGCGGCAGCCATCATGGC-3'
TDP43_Q331A Fw	5'-GCCGCCCAGGCAGCACTAGCTAGCAGTTGGGGTATGATG-3'
TDP43_Q331A Rv	5'-CATCATACCCCAACTGCTAGCTAGTGCTGCCTGGGCGGC-3'
TDP43_Q343A Fw	5'-ATGGGCATGTTAGCCAGCGCTCAGAACCAGTCAGGCCCATCG-3'
TDP43_Q343A Rv	5'-CGATGGGCCTGACTGGTTCTGAGCGCTGGCTAACATGCCCAT-3'
TDP43_L330A Fw	5'-GCTGCCGCCCAGGCAGCAGCTCAGAGCAGTTGGGGTATG-3'
TDP43_L330A Rv	5'-CATACCCCAACTGCTCTGAGCTGCTGCCTGGGCGGCAGC-3'
TDP43_W334A Fw	5'-GCAGCACTACAGAGCAGTGCTGGTATGATGGGCATGTTAG-3'
TDP43_W334A Rv	5'-CTAACATGCCCATCATACCAGCACTGCTCTGTAGTGCTGC-3'
TDP43_S333A Fw	5'-CAGGCAGCACTACAGAGCGCTTGGGGTATGATGGGCATG-3'
TDP43_S333A Rv	5'-CATGCCCATCATACCCCAAGCGCTCTGTAGTGCTGCCTG-3'
TDP43_L340A Fw	5'-TGGGGTATGATGGGCATGGCTGCCAGCCAGCAGAACCAG-3'
TDP43_L340A Rv	5'-CTGGTTCTGCTGGCTGGCAGCCATGCCCATCATACCCCA-3'

Supplementary Table 4. Details of the simulation setup. All simulations were performed at 150 mM NaCl. Starting and final configurations for all systems, which contains information on box dimensions, number of explicit water molecules, and ions, are also provided as Source Data.

System name	Box size (nm)	# of protein atoms	# of independent simulations	duration of each simulation (μs)	aggregate duration (μs)
Monomer	8.25 - 8.94	562	5	7.91 - 9.59	45.07
Dimer (AFv2.2)	8.00	1124	5	8.53 - 10.59	101.42
Dimer (AFv2.3)	9.77 - 10.66	1124	13	3.77 - 4.06	
Cross-linked dimer (S317C_SS)	7.36 - 8.13	1122	8	4.51 - 6.00	45.41
Trimer	8.83 - 9.91	1686	8	4.37 - 5.34	40.97
Tetramer (AFv2.2)	8.98	2248	2	1.40	2.80
Tetramer (AFv2.3)	8.92 - 9.63	2248	5	3.97 - 5.00	23.40
Tet-1	8.92	2248	50	0.1	5.0
Tet-2	9.63	2248	50	0.1	5.0
Hexamer	8.80 - 9.42	3372	5	4.59 - 5.30	25.69
Octamer	9.23 - 9.82	4496	4	4.89- 5.36	20.28
CTD full-length tetramer	18.00	7692	5	0.30	1.50

Supplementary Note 1

AUC Analysis of Designed Variants

Probing the assembly of TDP-43 CTD CR by biophysical techniques is complicated by aggregation at concentrations below those needed to saturate the CR multimerization by TDP-43 CTD wild-type. To drive TDP-43 CTD toward multimer formation, we formed disulfide cross-links (S-S) between engineered cysteine residues at S273C in TDP-43 CTD (with a sequence that is otherwise the wild-type sequence) (see Methods), as we previously used[10]. This cross-link is more than 40 residues away from the CR and previously we showed it results in chemical shift perturbations at all CR residue positions that mirror those shown for WT CTD with increasing concentrations, just with higher magnitude, suggesting similar interaction modes sampled by the TDP-43 CR[10]. At low concentrations (~5 μM), AUC experiments for the cross-linked variant show a sedimentation coefficient consistent with a dimer form. (**Supplementary Fig. 7A**). At ~11 μM and above a species with sedimentation coefficients consistent with a tetramer or higher is also observed, consistent with cooperative assembly (**Supplementary Fig. 7A**). However, at these concentrations, the samples show evidence of aggregation by AUC experiments, as we previously observed for NMR sample[10] and the low solubility of TDP-43 CTD S273C cross-linked samples hinders further characterization of the multimeric state using these samples.

To overcome low solubility, we engineered a new CTD variant by introducing charged, glutamic acid residues at serine positions (S→E) in the disordered flanking regions of CTD, mimicking how phosphorylation may counteract pathological aggregation, enhance the liquidity and dynamic nature of TDP-43 condensates, while preserving nuclear import or RNA regulatory functions.[11,12] (See Table S1). This modification significantly enhanced solubility while preserving the helical structure in the CR structure, as indicated by NMR spectral fingerprint of the monomeric S→E variant (**Supplementary Fig. 6**), suggesting that this variant should not disrupt helical multimerization. However, placing the cross-link position in the S→E variant at residue 273 (S→E S273C cross-linked) resulted in no apparent self-assembly and small chemical

shift perturbations (**Supplementary Fig. 7B,E**), suggesting that electrostatic repulsion discourages self-interactions. Reasoning that cooperative CR-CR multimerization in the S→E variants could be enhanced by placing the cross-link position closer to the CR, we designed two obligate dimers with cross-linking site closer to CR at position S305 and S317 (S→E S305C and S→E S317C). To demonstrate that cross-linking closer to the CR does not alter the CR structure, we simulated the cross-linked dimer, TDP-$43_{310\text{-}350}$ S317C S-S, by mutating S317 to cysteine in each chain of AF2-Multimer dimer models using Chimera[13] and adding a disulfide bond (S-S) between cysteine residues using the pdb2gmx -ss command. Simulations of the S317C cross-linked dimer (total time ~45 μs) show that per-residue helix fractions and pairwise intermolecular contacts for the cross-linked dimer ensemble remained similar to WT dimer (**Supplementary Fig. 8**), suggesting that the fundamental features of CR remain intact.

While AUC of the S→E S273C dimers indicate mostly dimer species at all concentrations studied, with traces (<1%) of a higher-order tetramer (**Supplementary Fig. 7B**), cross-linking closer to the CR (S→E S305C and S→E S317C) enhances the assembly. The sedimentation coefficient distributions for S→E S317C at different concentrations show the presence of two main peaks, corresponding to the dimer (~2.4 S) and tetramer (~3.6 S) states, with peaks of the latter are more pronounced at higher concentrations (**Supplementary Fig. 7C, right**). The profiles for S→E S305C indicate peaks corresponding to dimer and tetramer as well as octamer (~4.5 S), with the fraction of the octamer increasing with protein concentration (**Supplementary Fig. 7C, left**). The key observation is that analysis of the weighted-average sedimentation coefficient isotherms suggest a dimer-tetramer self-association K_d of 46 μM for S→E S317C and 58 μM for S→E S305C (**Supplementary Fig. 9A,B**), consistent with our expectation that obligate dimers formed by cross-linking enhance tetramer formation, bypassing the barrier for weak dimer assembly (with mM K_d) seen for the (non-crosslinked) wild-type. To demonstrate that higher-order assemblies are mediated by the helical region, we studied the multimeric size distributions of the helix-breaking mutant, A326P in the S→E S305C dimer. SV-AUC data shows the absence of the higher-order structures, with only

peaks for a dimer state, suggesting that the helical region is essential for observed multimerization (**Supplementary Fig. 7D**).

Supplementary Note 2

NMR Analysis of Designed Variants

After confirming the presence of higher-order multimers larger than dimer, we used NMR to characterize the structural changes upon helix-mediated TDP-43 CR multimerization using the cross-linked S→E dimers. The 2D NMR spectrum of S→E dimers with cross-linking positions show chemical shift differences in the CR that increase as the cross-link is moved closer to the CR and lie along the same line formed by WT CTD suggesting transient formation of helix-helix dimers increases as the cross-linking distance to the CR is shortened (**Supplementary Fig. 7E**). Large CSPs for CR residues as the protein concentration increases, especially for S305C and S317C, suggest multimerization beyond dimer occurs in this concentration range, consistent with AUC experiments. Importantly, these chemical shift perturbations lie along a new vector, consistent with the formation of a multimeric structure distinct from the dimer (**Supplementary Fig. 7E**)[6]. CR peaks broadened at higher concentrations, indicating the increasing population of the assembled state. Comparatively, the helix-breaking mutant, S→E A326P S305C dimer does not show CSPs up to 60 μM protein concentrations (**Supplementary Fig. 7E**), confirming that higher-order multimers, larger than dimer, observed via SV-AUC measurements, are mediated by TDP-43 CR.

To assess the impact of assembly on molecular structure and motions, we selected S→E S305C dimers for NMR measurements (at 47 ºC to improve NMR spectra for CR residues). ^{15}N chemical shift differences between the cross-linked (oxidized) and non-cross-linked (reduced) S305C variants showed upfield (negative) CSPs for residues 316-341 (**Supplementary Fig. 7F**), consistent with enhanced helix-helix contacts and increased helicity[6,14]. In the cross-linked S→E S305C dimers, pronounced positive increases in ^{13}C secondary shifts were observed for the entire CR (**Supplementary Fig. 7G**), suggesting enhanced helicity upon multimerization in both the main helical region (aa: 320-331) and the subsequent region (aa: 332-343). ^{15}N spin relaxation experiments

revealed slowed backbone motions for the entire CR upon multimerization with transverse relaxation rate constants (^{15}N R_2) showing around a four-fold increase compared to monomeric controls (**Supplementary Fig. 7H**). This indicates slowed motions across residues 315–343 upon helix-helix assembly, consistent with the formation of locally rigid, higher-order structure within CR.

Supplementary Note 3

Structural model of the tetrameric helical assembly of TDP-43 CTD

We and others have demonstrated the critical role of flanking region residues in CTD self-assembly[15-17]. Our recent work showed that CTD self-assembly is driven by helix-mediated CR:CR interactions, strengthened by transient interactions involving the flanking disordered regions, with aromatic and methionine residues playing key roles[15]. To investigate the role of flanking IDR residues in the tetrameric model, the full-length CTD tetramer structure was prepared by using the Tet-1 model for CR residues and modeling the remaining CTD residues with MODELLER[4] (**Supplementary Fig. 12A**). A 300 ns coarse-grained (CG) simulation was then performed using the MODELLER-generated structure, keeping the CR residues fixed as a rigid body in the Tet-1 conformation while allowing the IDR regions to relax, following our previous protocol[18]. This pre-equilibration step was essential for refining the IDR regions and ensuring that subsequent AA simulations could be conducted within a reasonably sized simulation box. For this, we used the HPS-Urry model[19], which we previously demonstrated to be effective in capturing both the monomeric ensemble and the phase separation propensity of the CTD[15]. Following the CG simulation, the TTClust clustering method[20] was applied to identify the 5 most representative structures of the full-length CTD tetramer for subsequent AA simulations (**Supplementary Fig. 12B**). These CG structures were then back-mapped to atomistic representations using MODELLER[4], and 5 independent AA simulations were set up using each of these back-mapped structures. In full-length CTD tetramer simulations, the CR helicity and CR:CR tetrameric interfacial contacts remained stable (**Supplementary Fig. 12C,D**), confirming the structural stability of the helical CR tetramer structure within full-length CTD. Residues within the CR, including M322, A326,

A329, L330, S333, M336/337, and L340, maintained high intermolecular contacts, consistent with shorter fragment simulations of Tet-1 (**Supplementary Fig. 12E, F**).

Supplementary References:

1 Bolognesi, B. *et al.* The mutational landscape of a prion-like domain. *Nat Commun* **10**, 4162 (2019). https://doi.org:10.1038/s41467-019-12101-z

2 Schuck, P. On the analysis of protein self-association by sedimentation velocity analytical ultracentrifugation. *Analytical Biochemistry* **320**, 104-124 (2003). https://doi.org:https://doi.org/10.1016/S0003-2697(03)00289-6

3 Brautigam, C. A. Calculations and Publication-Quality Illustrations for Analytical Ultracentrifugation Data. *Methods Enzymol* **562**, 109-133 (2015). https://doi.org:10.1016/bs.mie.2015.05.001

4 Eswar, N. *et al.* Comparative protein structure modeling using Modeller. *Curr Protoc Bioinformatics* **Chapter 5**, Unit-5.6 (2006). https://doi.org:10.1002/0471250953.bi0506s15

5 Vögeli, B., Ying, J., Grishaev, A. & Bax, A. Limits on Variations in Protein Backbone Dynamics from Precise Measurements of Scalar Couplings. *Journal of the American Chemical Society* **129**, 9377-9385 (2007). https://doi.org:10.1021/ja070324o

6 Conicella, Alexander E., Zerze, Gül H., Mittal, J. & Fawzi, Nicolas L. ALS Mutations Disrupt Phase Separation Mediated by α-Helical Structure in the TDP-43 Low-Complexity C-Terminal Domain. *Structure* **24**, 1537-1549 (2016). https://doi.org:10.1016/j.str.2016.07.007

7 Shen, Y. & Bax, A. SPARTA+: a modest improvement in empirical NMR chemical shift prediction by means of an artificial neural network. *Journal of Biomolecular NMR* **48**, 13-22 (2010). https://doi.org:10.1007/s10858-010-9433-9

8 Kabsch, W. & Sander, C. Dictionary of protein secondary structure: Pattern recognition of hydrogen-bonded and geometrical features. *Biopolymers* **22**, 2577-2637 (1983). https://doi.org:https://doi.org/10.1002/bip.360221211

9 Camilloni, C., De Simone, A., Vranken, W. F. & Vendruscolo, M. Determination of secondary structure populations in disordered states of proteins using nuclear magnetic resonance chemical shifts. *Biochemistry* **51**, 2224-2231 (2012).

10 Conicella, A. E. *et al.* TDP-43 α-helical structure tunes liquid–liquid phase separation and function. *Proceedings of the National Academy of Sciences* **117**, 5883-5894 (2020). https://doi.org:10.1073/pnas.1912055117

11 Gruijs da Silva, L. A. *et al.* Disease-linked TDP-43 hyperphosphorylation suppresses TDP-43 condensation and aggregation. *EMBO J* **41**, e108443 (2022). https://doi.org:10.15252/embj.2021108443

12 Monahan, Z. *et al.* Phosphorylation of the FUS low-complexity domain disrupts phase separation, aggregation, and toxicity. *EMBO J* **36**, 2951-2967 (2017). https://doi.org:10.15252/embj.201696394

13 Pettersen, E. F. *et al.* UCSF Chimera—a visualization system for exploratory research and analysis. *Journal of computational chemistry* **25**, 1605-1612 (2004).

14 Wang, Y. & Jardetzky, O. Probability-based protein secondary structure identification using combined NMR chemical-shift data. *Protein Science* **11**, 852-861 (2002). https://doi.org:https://doi.org/10.1110/ps.3180102

15 Mohanty, P. *et al.* A synergy between site-specific and transient interactions drives the phase separation of a disordered, low-complexity domain. *Proceedings of the National Academy of Sciences* **120**, e2305625120 (2023).

16 Li, H. R., Chiang, W. C., Chou, P. C., Wang, W. J. & Huang, J. R. TAR DNA-binding protein 43 (TDP-43) liquid-liquid phase separation is mediated by just a few aromatic residues. *J Biol Chem* **293**, 6090-6098 (2018). https://doi.org:10.1074/jbc.AC117.001037

17 Schmidt, H. B., Barreau, A. & Rohatgi, R. Phase separation-deficient TDP43 remains functional in splicing. *Nature Communications* **10**, 4890 (2019). https://doi.org:10.1038/s41467-019-12740-2

18 Mammen Regy, R., Zheng, W. & Mittal, J. in *Methods in Enzymology* Vol. 646 (ed Christine D. Keating) 1-17 (Academic Press, 2021).

19 Regy, R. M., Thompson, J., Kim, Y. C. & Mittal, J. Improved coarse-grained model for studying sequence dependent phase separation of disordered proteins. *Protein Sci* **30**, 1371-1379 (2021). https://doi.org:10.1002/pro.4094

20 Tubiana, T., Carvaillo, J. C., Boulard, Y. & Bressanelli, S. TTClust: A Versatile Molecular Simulation Trajectory Clustering Program with Graphical Summaries. *J Chem Inf Model* **58**, 2178-2182 (2018). https://doi.org:10.1021/acs.jcim.8b00512

Unprocessed, uncropped scans for Supplementary Figure 4:

WT, Q331A, W334A – Lamin A/C

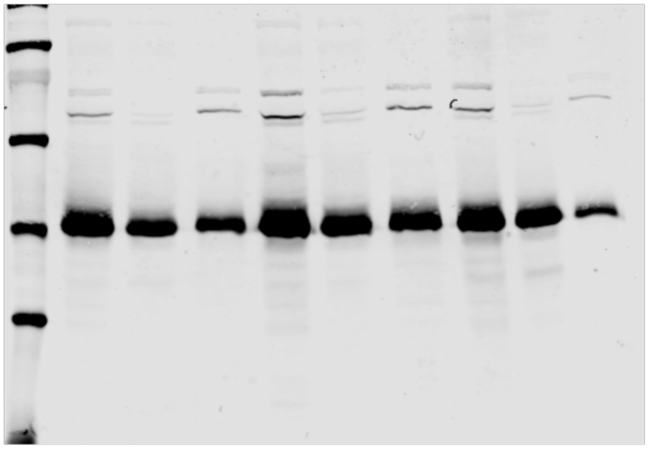

WT, Q331A, W334A – Tubulin

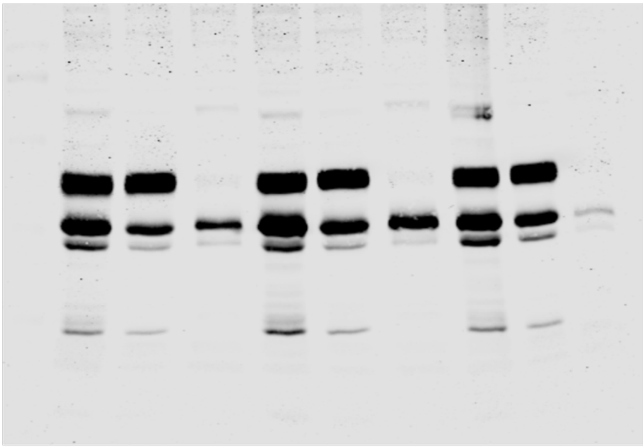

L330 – Lamin A/C

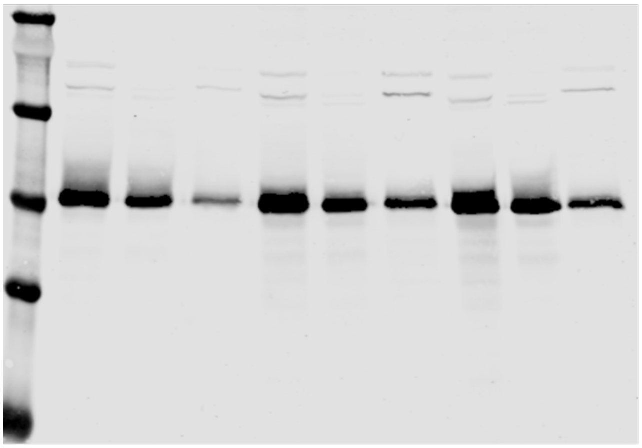

L330 – tubulin

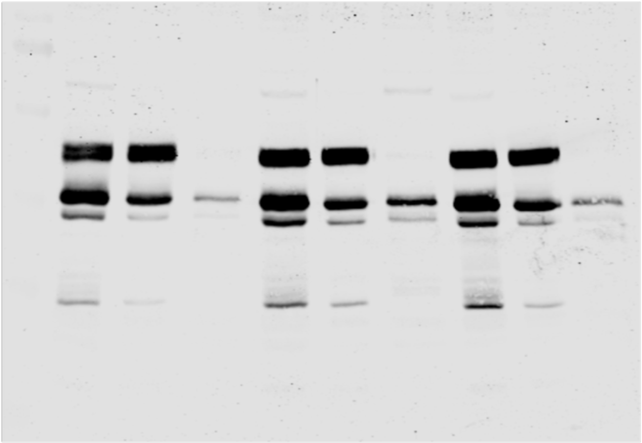

WT, Q327A, Q343A – lamin A/C

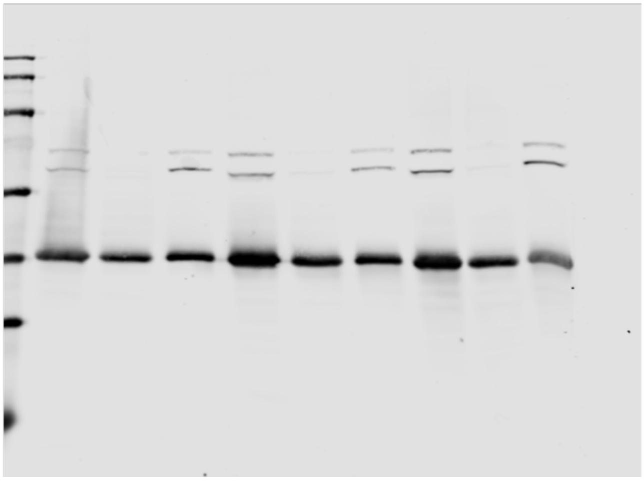

WT, Q327A, Q343A – tubulin

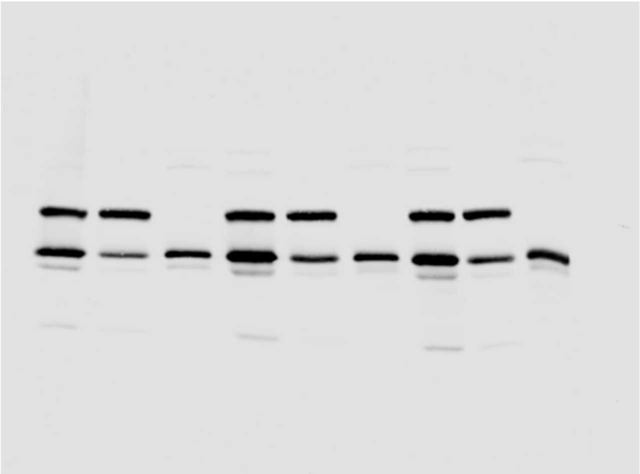

S333A, L340A – lamin A/C

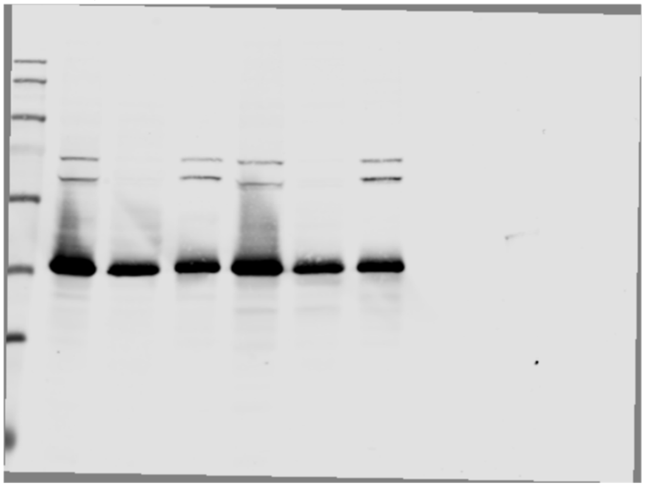

S333A, L340A – tubulin

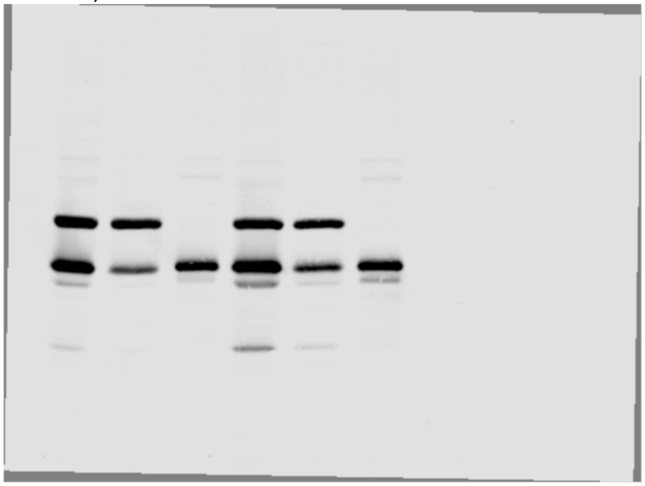

WT

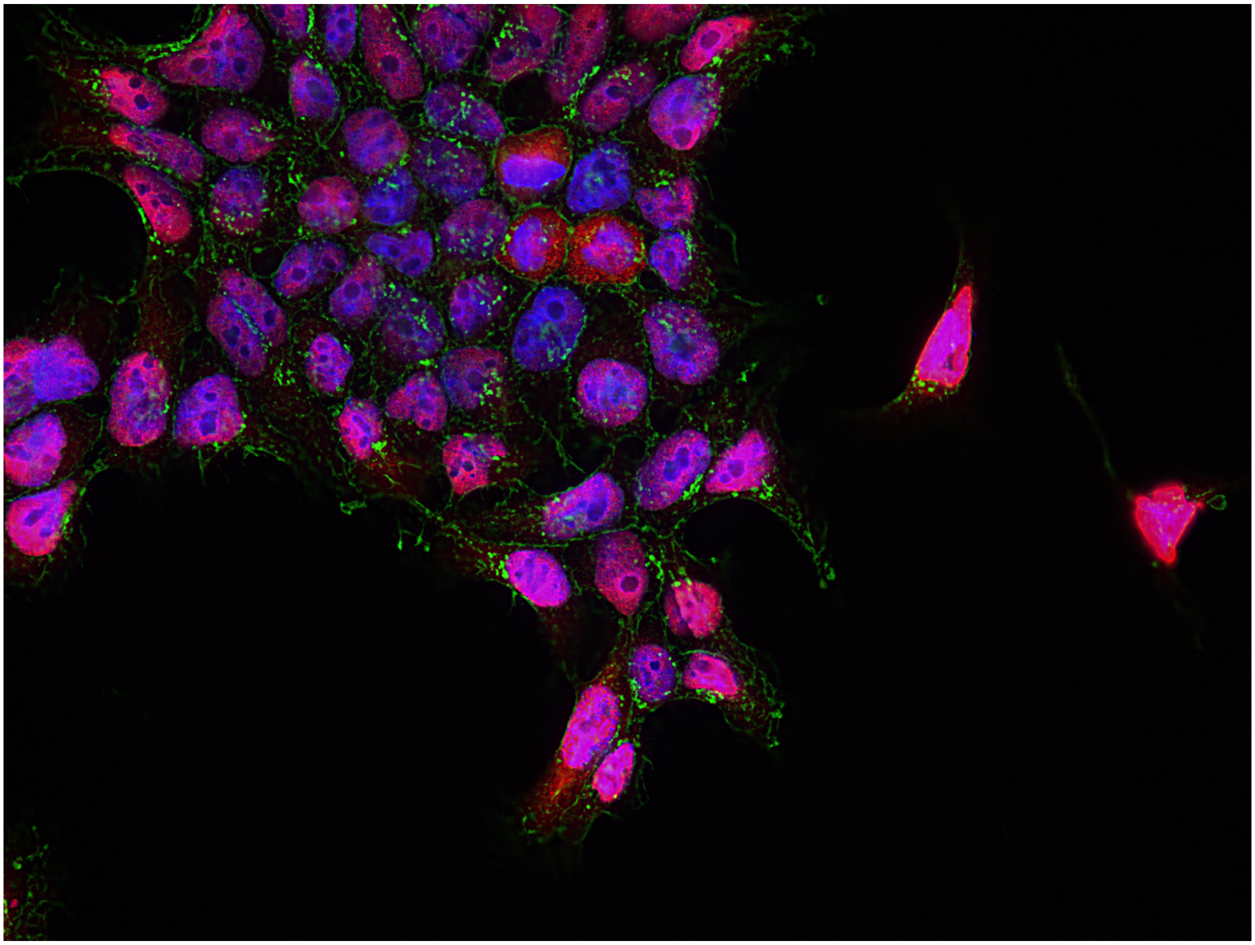

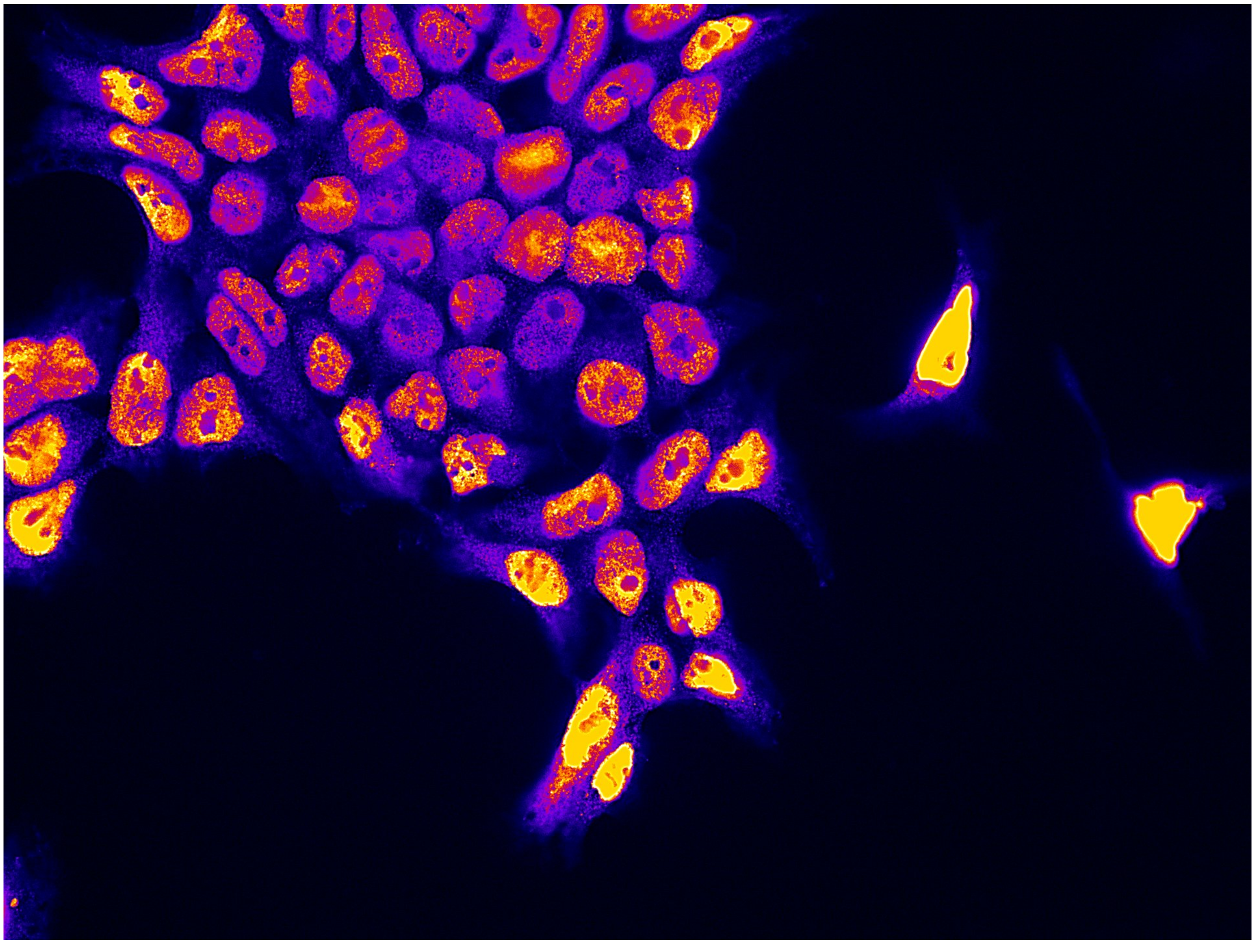

A326P

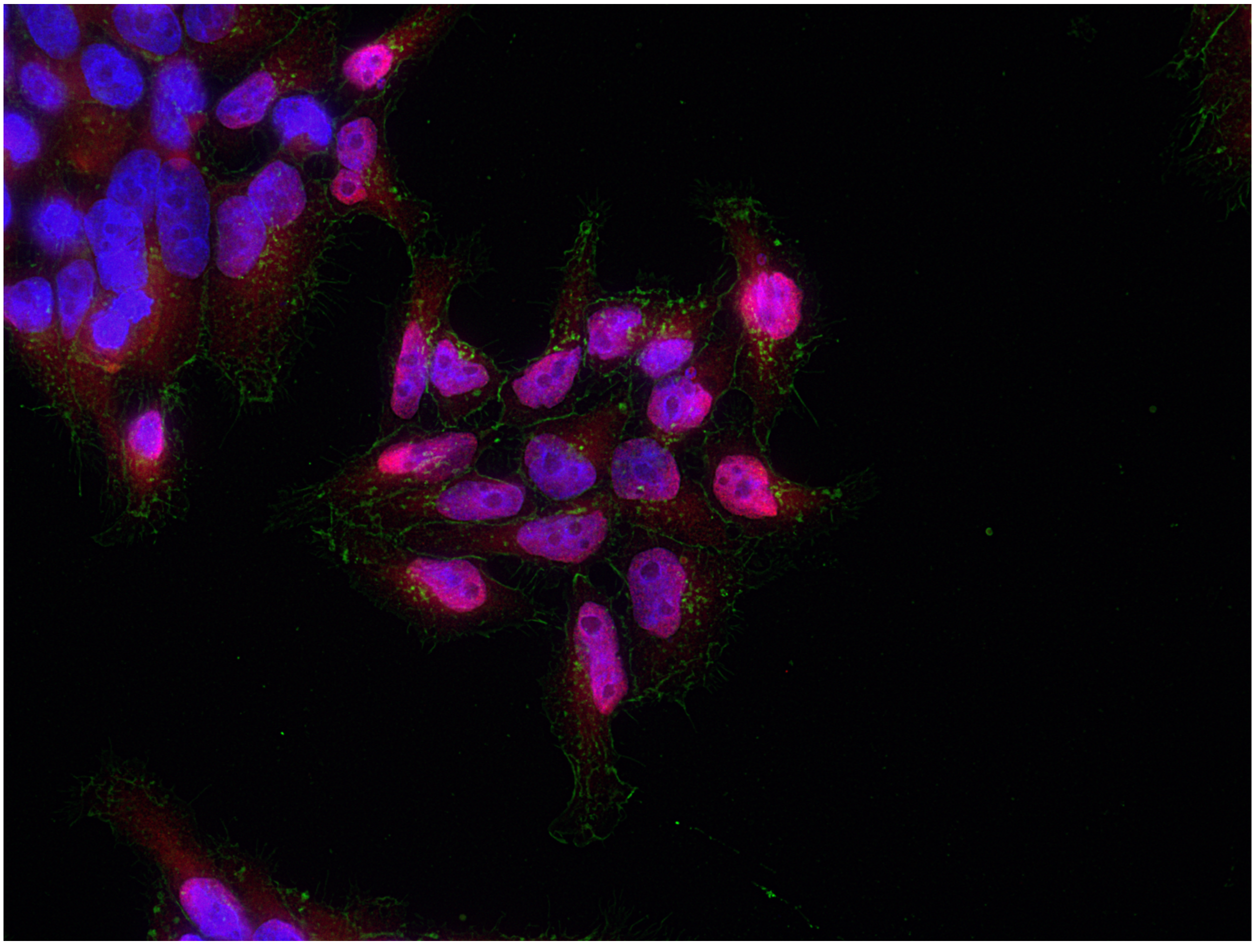

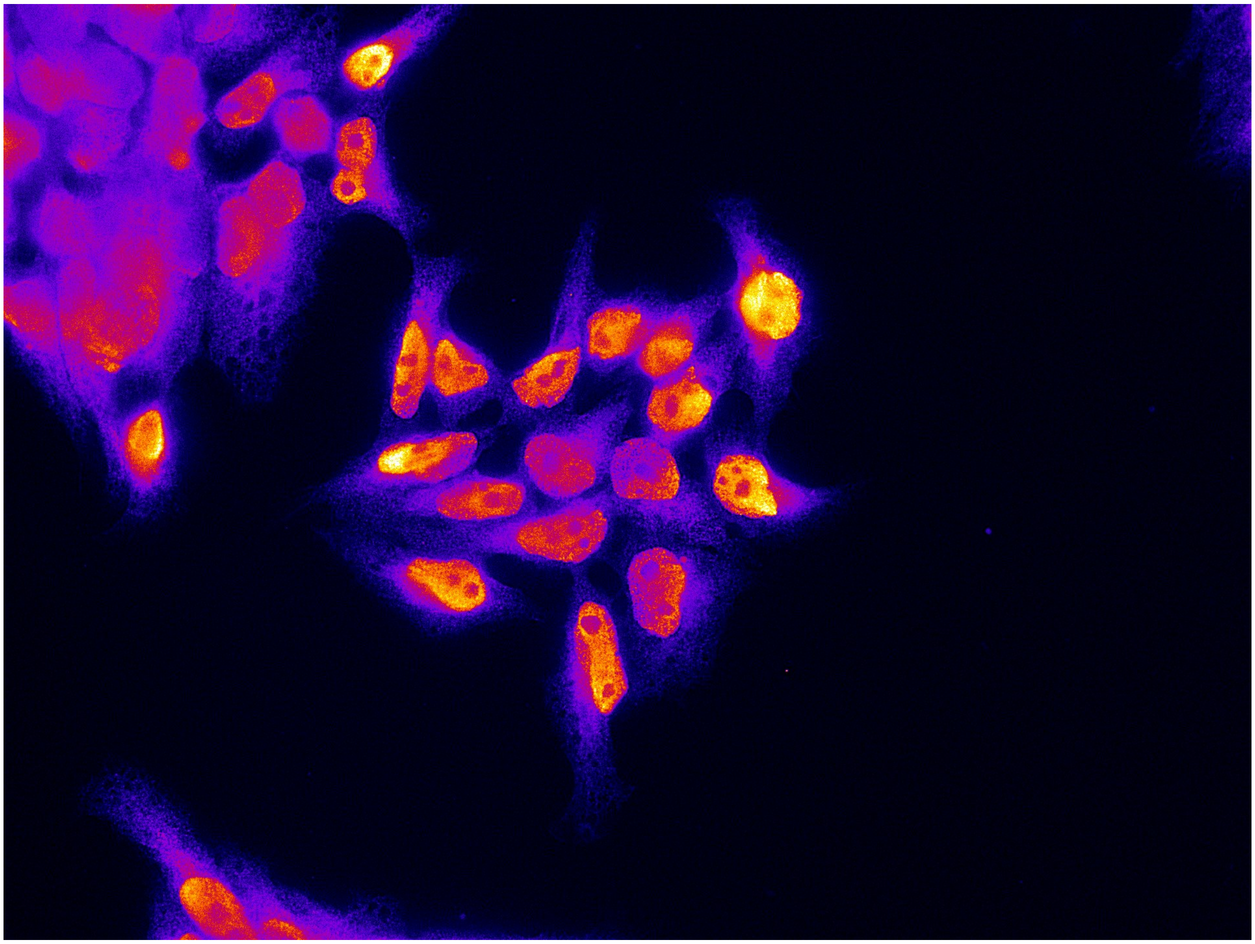

L330A

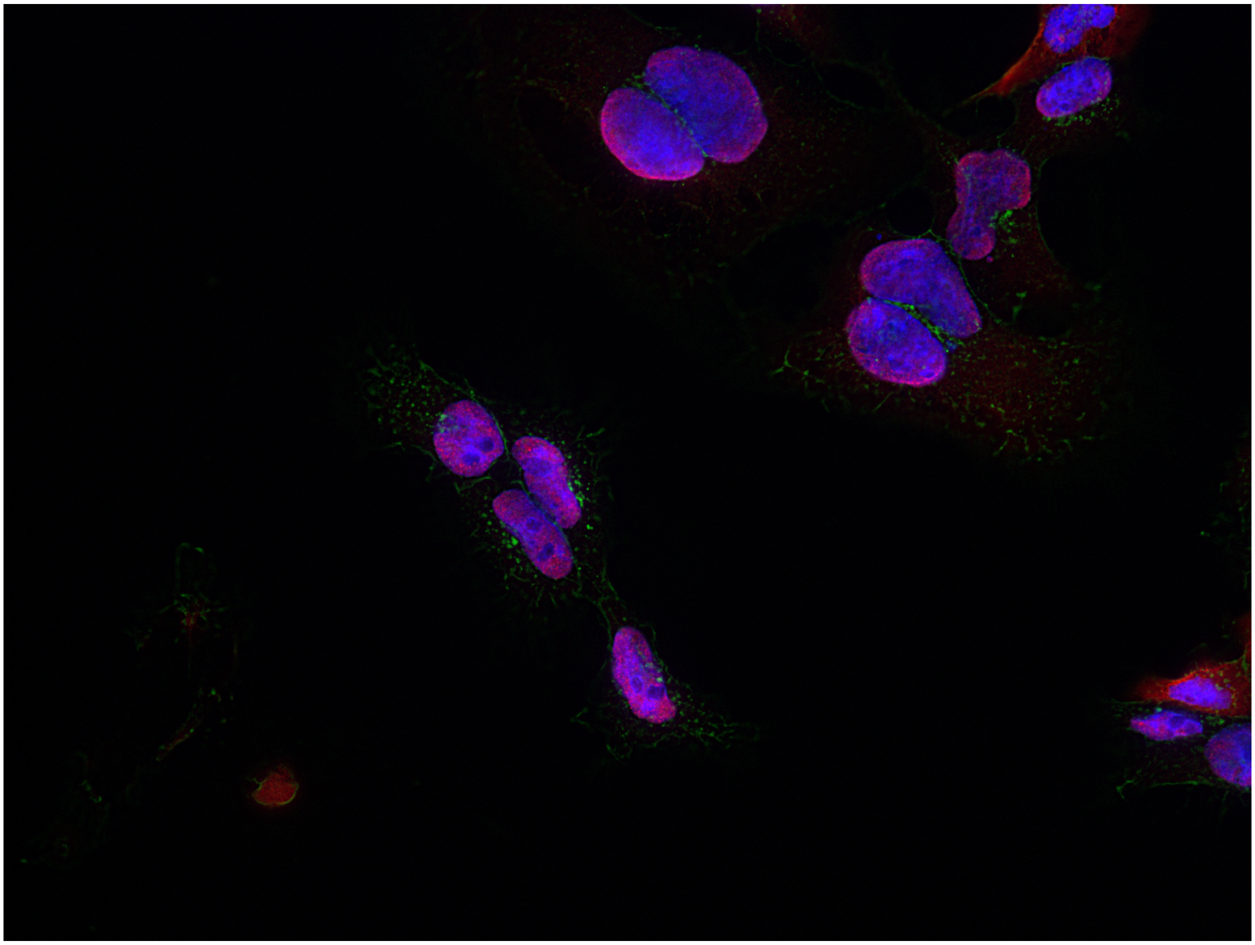

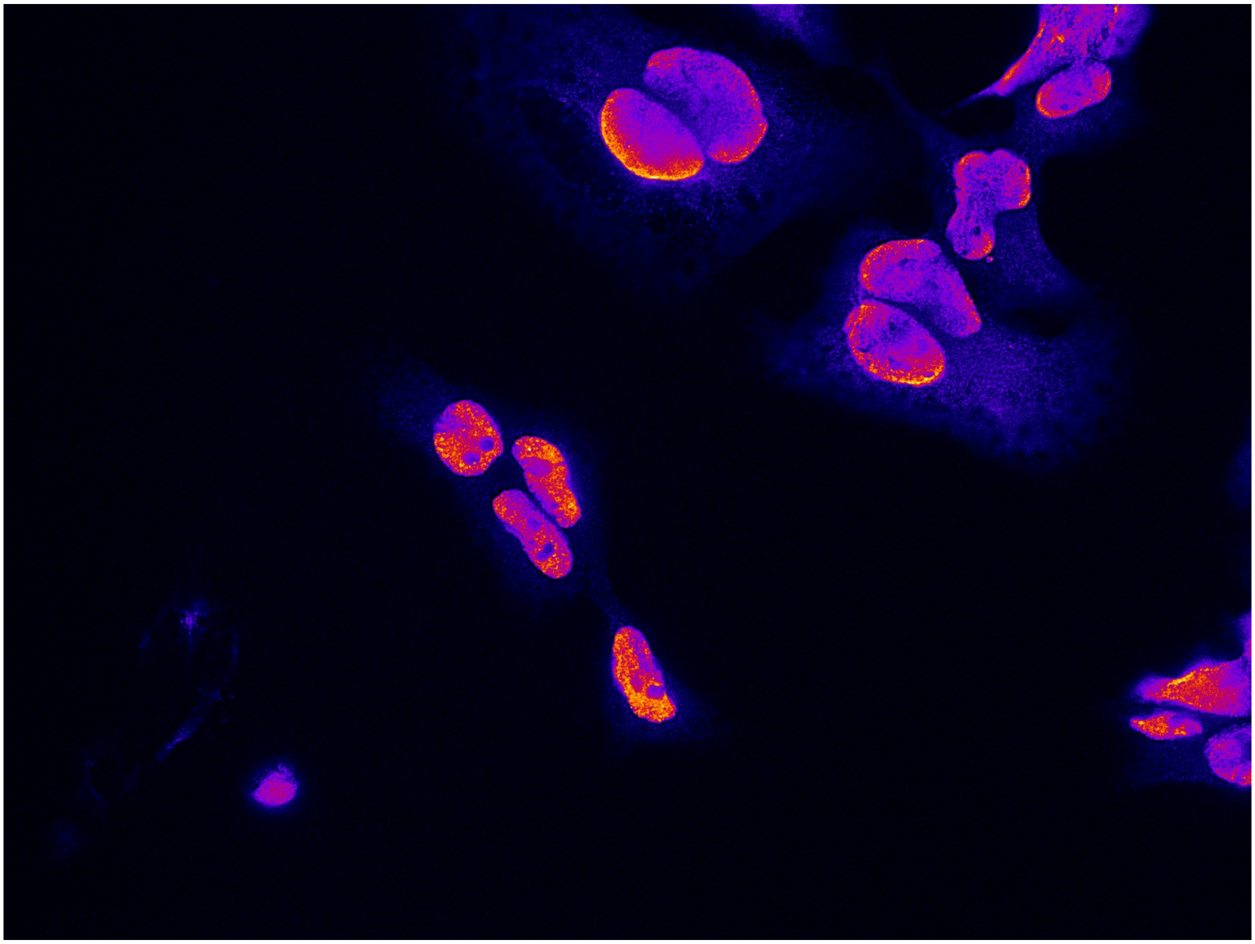

S333A

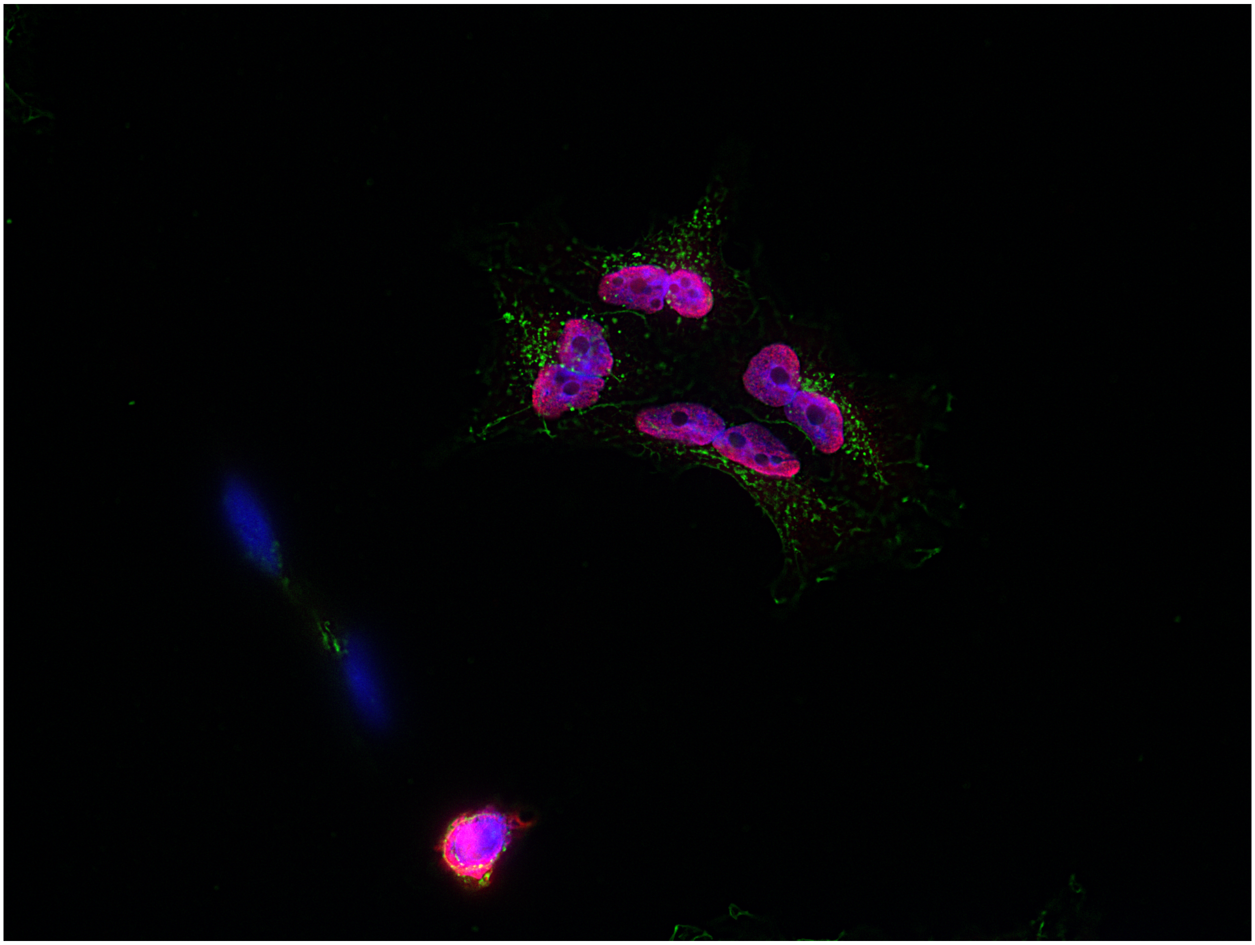

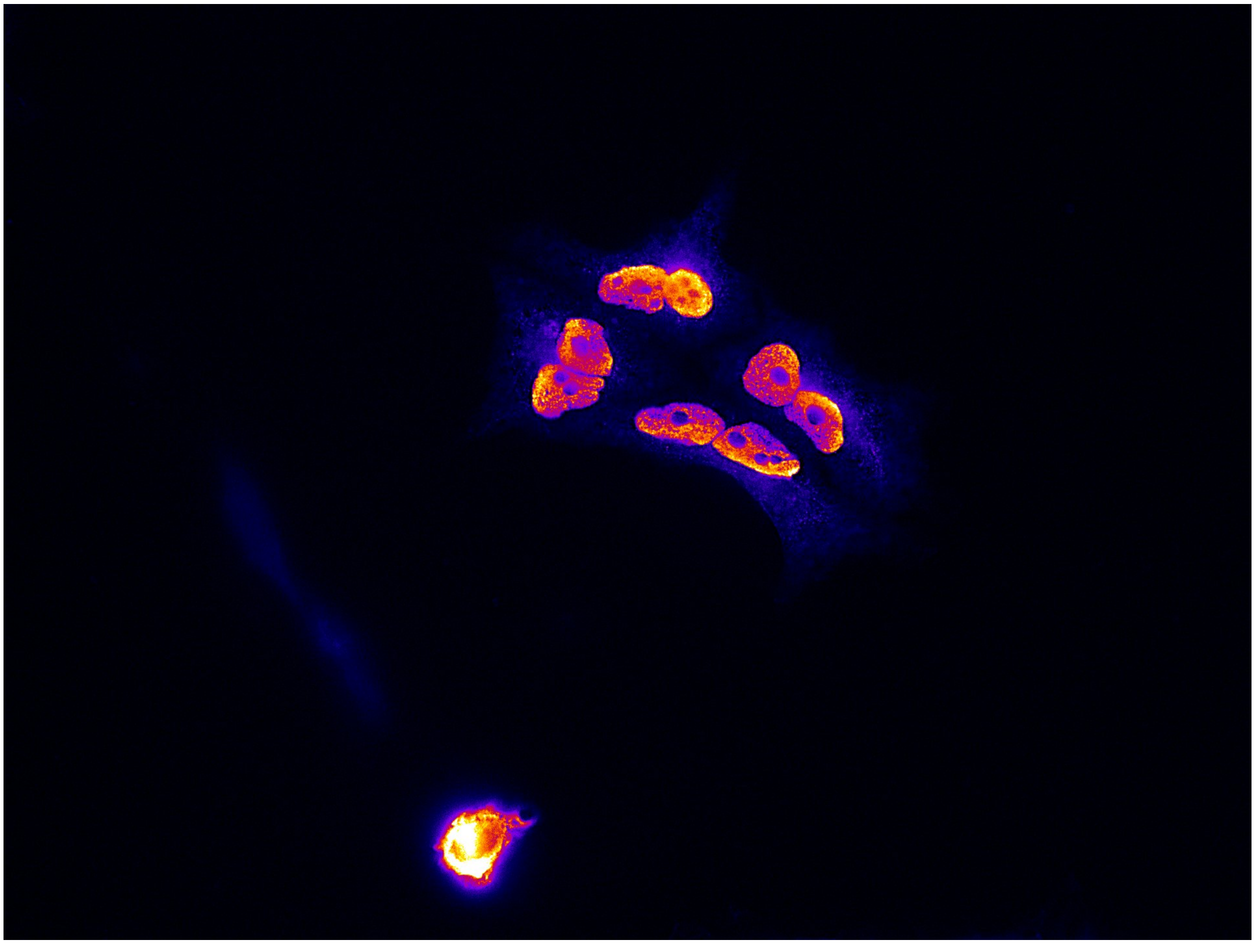

W334A

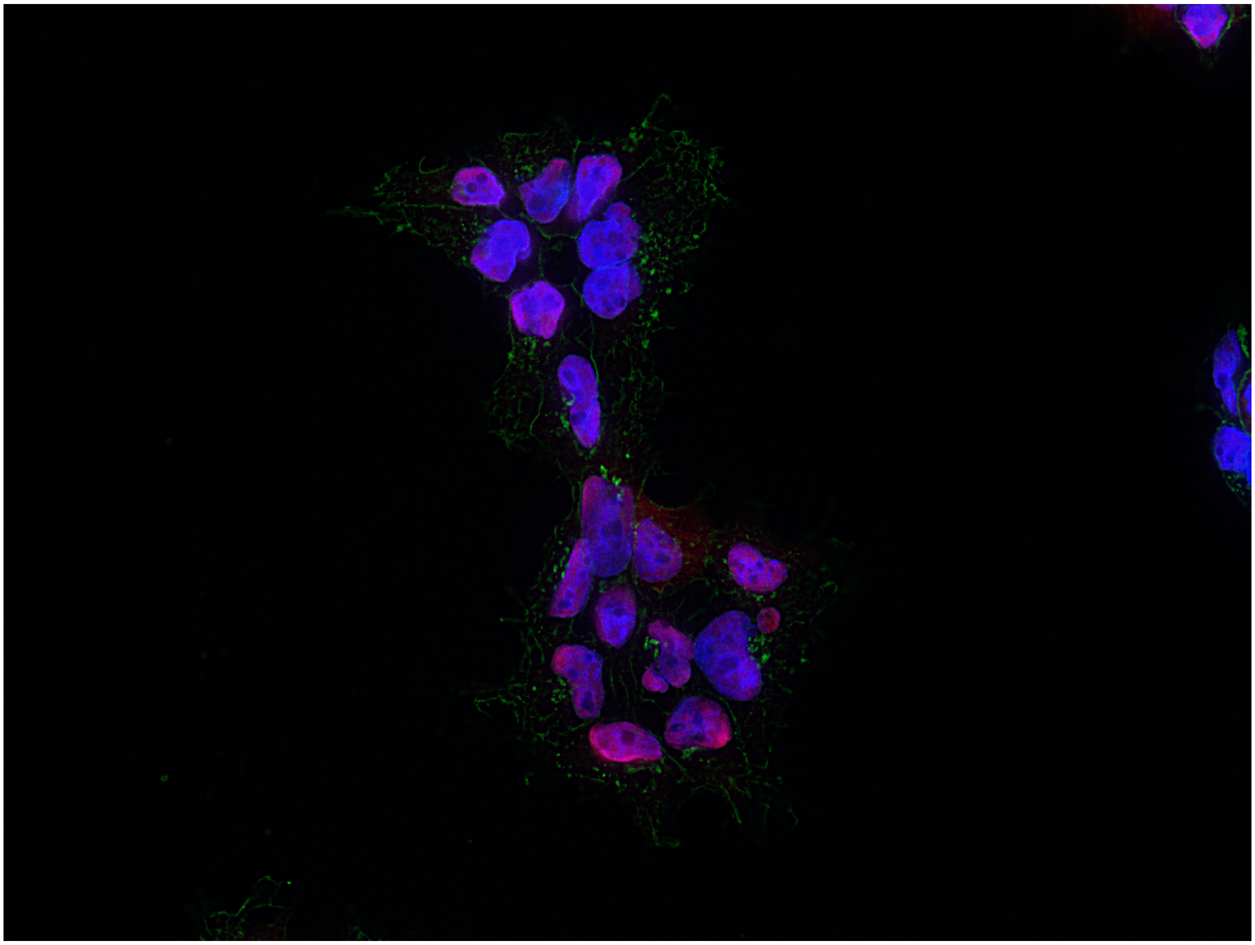

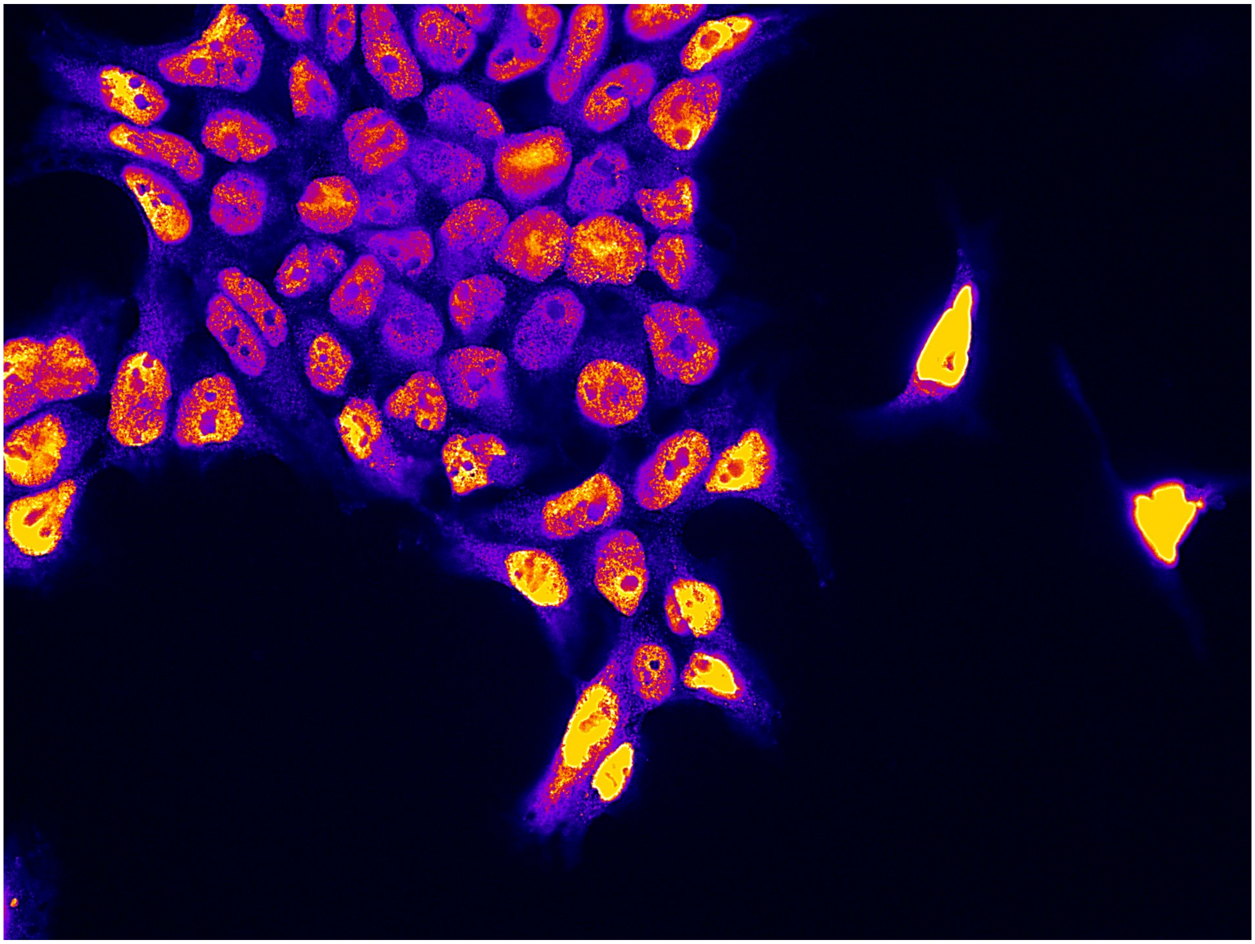

Supplement: Supplementary file 1 — Supplementary Information [file 41467_2025_65546_MOESM1_ESM.pdf]
