# Supplementary material for: Structural details of helix-mediated multimerization of the conserved region of TDP-43 C-terminal domain
Source: Nat Commun. 2025 Nov 26;16:10528. doi: 10.1038/s41467-025-65546-w (PMC12658102; doi:10.1038/s41467-025-65546-w)
Supplement: Supplementary file 2 — Reporting Summary [file 41467_2025_65546_MOESM2_ESM.pdf]

nature portfolio

Corresponding author(s): Nicolas Fawzi, Jeetain Mittal

Last updated by author(s): Oct 2, 2025

Reporting Summary

Nature Portfolio wishes to improve the reproducibility of the work that we publish. This form provides structure for consistency and transparency in reporting. For further information on Nature Portfolio policies, see our Editorial Policies and the Editorial Policy Checklist.

Statistics

For all statistical analyses, confirm that the following items are present in the figure legend, table legend, main text, or Methods section.

n/a	Confirmed	
☐	☒	The exact sample size (n) for each experimental group/condition, given as a discrete number and unit of measurement
☐	☒	A statement on whether measurements were taken from distinct samples or whether the same sample was measured repeatedly
☐	☒	The statistical test(s) used AND whether they are one- or two-sided<br>*Only common tests should be described solely by name; describe more complex techniques in the Methods section.*
☒	☐	A description of all covariates tested
☐	☒	A description of any assumptions or corrections, such as tests of normality and adjustment for multiple comparisons
☐	☒	A full description of the statistical parameters including central tendency (e.g. means) or other basic estimates (e.g. regression coefficient) AND variation (e.g. standard deviation) or associated estimates of uncertainty (e.g. confidence intervals)
☐	☒	For null hypothesis testing, the test statistic (e.g. F, t, r) with confidence intervals, effect sizes, degrees of freedom and P value noted<br>*Give P values as exact values whenever suitable.*
☒	☐	For Bayesian analysis, information on the choice of priors and Markov chain Monte Carlo settings
☒	☐	For hierarchical and complex designs, identification of the appropriate level for tests and full reporting of outcomes
☐	☒	Estimates of effect sizes (e.g. Cohen's d, Pearson's r), indicating how they were calculated

Our web collection on statistics for biologists contains articles on many of the points above.

Software and code

Policy information about availability of computer code

Data collection	NMR experiments were recorded on on a Bruker Avance III HD HD 850 MHz spectrometer. All atom simulations were run using Amber22 with the Amber03ws force-field (https://doi.org/10.5281/zenodo.17059939) and solvated with TIP4P/2005 water model. Installation of AlphaFold-Multimer (version 2.2.0/2.3.0) on Grace cluster (Texas A&M University HPRC) was used for multimer predictions.
Data analysis	Matlab R2023a was used for data analysis. NMR data was analyzed using nmrpipe and CCPNMR 2.5.2. Micrographs were processed using ImageJ. Simulation analyses performed with GROMACS version 2021.6. Codes to run and analyze atomistic simulations are available publicly and can be found at https://ambermd.org/, https://gromacs.org/, https://www.mdtraj.org/ and https://www.mdanalysis.org/. All snapshots from atomistic simulations were generated using UCSF Chimera version 1.18/ChimeraX version 1.8. For preparation and analysis of the analytical ultracentrifugation (AUC) data, publicly available software was used and can be found at REDATE Version 1.01 (https://www.utsouthwestern.edu/research/core-facilities/mbr/software/), SEDFIT Version 16.1c (https://sedfitsedphat.nibib.nih.gov/software), SEDPHAT Version 15.2b (https://sedfitsedphat.nibib.nih.gov/software), GUSSI Version 2.10 (https://www.utsouthwestern.edu/research/core-facilities/mbr/software/), SEDNTERP Version 3.0.4 (http://www.jphilo.mailway.com/sednterp.htm).

For manuscripts utilizing custom algorithms or software that are central to the research but not yet described in published literature, software must be made available to editors and reviewers. We strongly encourage code deposition in a community repository (e.g. GitHub). See the Nature Portfolio guidelines for submitting code & software for further information.

Data

Policy information about availability of data

All manuscripts must include a data availability statement. This statement should provide the following information, where applicable:

- Accession codes, unique identifiers, or web links for publicly available datasets
- A description of any restrictions on data availability
- For clinical datasets or third party data, please ensure that the statement adheres to our policy

Data Availability
Sequences for proteins used in this work are included in the Supplementary Information file. Plasmids for these sequences are available from Addgene https://www.addgene.org/Nicolas_Fawzi/. NMR chemical shift assignments for the TDP-43 C-terminal domain residues 300-360 at pH 7 are deposited with the Biological Magnetic Resonance Data Bank (BMRB) entry 53381 [https://dx.doi.org/10.13018/BMR 53381]. Time domain and processed NMR data are deposited at BMRbig with entry identifier bmrbig135 [https://bmrbig.org/released/bmrbig135]. BMRB entry 26823 [https://dx.doi.org/10.13018/BMR26823] from previously published studies16 was used in this work. AlphaFold2-Multimer–predicted structures used in the AAMD simulations (in PDB format), together with their corresponding .pkl files containing pLDDT and PAE information, have been deposited on Zenodo [https://doi.org/10.5281/zenodo.17059939] (ref.89). Simulation input files, along with the starting and final configurations for all atomistic simulations, have been deposited on Zenodo [https://doi.org/10.5281/zenodo.17059939] (ref.89). Unless otherwise stated, all data supporting the results of this study can be found in the article, supplementary, and source data files. Source data are provided with this publication. Other materials are available upon reasonable request to the corresponding authors.

Research involving human participants, their data, or biological material

Policy information about studies with human participants or human data. See also policy information about sex, gender (identity/presentation), and sexual orientation and race, ethnicity and racism.

Reporting on sex and gender	n/a
Reporting on race, ethnicity, or other socially relevant groupings	n/a
Population characteristics	n/a
Recruitment	n/a
Ethics oversight	n/a

Note that full information on the approval of the study protocol must also be provided in the manuscript.

Field-specific reporting

Please select the one below that is the best fit for your research. If you are not sure, read the appropriate sections before making your selection.

☒ Life sciences ☐ Behavioural & social sciences ☐ Ecological, evolutionary & environmental sciences

For a reference copy of the document with all sections, see nature.com/documents/nr-reporting-summary-flat.pdf

Life sciences study design

All studies must disclose on these points even when the disclosure is negative.

Sample size	Biochemical assays were conducted in two biological replicates (independently expressed and purified protein).<br>Biochemical assays were conducted with three technical replicates for calculation of statistics. This number is selected due to the high reproducibility of this assay type in previous studies (PMID: 27545621, PMID: 32132204).<br>The reproducibility of the cell assays was verified by conducting at least five independent replicates for each mutant cell line. Mutant function was compared in parallel to wild-type for each experiment. Wild type function showed no significant variation. In addition, we measured activity using different batches of freshly thawed stable cell lines from low passage number stocks.
Data exclusions	No data were excluded.
Replication	Biological replicates of all experiments were performed with independent protein preparations.
Randomization	Due to the nature of the study, randomization of experiments were not required.
Blinding	Blinding was not used as no experimenter-judgement was used in measurements.

Reporting for specific materials, systems and methods

We require information from authors about some types of materials, experimental systems and methods used in many studies. Here, indicate whether each material, system or method listed is relevant to your study. If you are not sure if a list item applies to your research, read the appropriate section before selecting a response.

Materials & experimental systems

n/a	Involved in the study
☐	☒ Antibodies
☐	☒ Eukaryotic cell lines
☒	☐ Palaeontology and archaeology
☒	☐ Animals and other organisms
☒	☐ Clinical data
☒	☐ Dual use research of concern
☒	☐ Plants

Methods

n/a	Involved in the study
☒	☐ ChIP-seq
☒	☐ Flow cytometry
☒	☐ MRI-based neuroimaging

Antibodies

Antibodies used

TDP-43 antibody anti-rabbit (Dilution – 1:4000 Proteintech, cat. 10782-2-AP, lot. 00132288)
HA antibody anti-mouse (Dilution - 1:200, Cell signaling, cat. 2367S, lot. 5)
Alexa fluor 555 donkey anti-mouse (Dilution – 1: 1000,Invitrogen, cat. A31570, lot.1270147)

Validation

Antibodies for TDP-43 were validated by immunoblotting and immunofluorescence of cells in which TARDBP expression was downregulated by RNAi, compared to control treated cells (non-targeting siRNA) (PMID: 28167528). The HA antibody was similarly validated with cells induced to express HA-tagged constructs and compared to cells non-induced cells.

Eukaryotic cell lines

Policy information about cell lines and Sex and Gender in Research

Cell line source(s)

Flp-In™ T-REx™ 293 Cell Line (Thermo Fisher, cat. R78007)

Authentication

genotyping and karyotyping prior to to use

Mycoplasma contamination

All cell lines tested negative for mycoplasma contamination using available commercial kits.

Commonly misidentified lines
(See ICLAC register)

These lines are fully identified from a commercial source.

Plants

Seed stocks

n/a

Novel plant genotypes

n/a

Authentication

n/a
